# Abscisic acid signaling gates salt-induced responses of plant roots

**DOI:** 10.1073/pnas.2406373122

**Published:** 2025-02-05

**Authors:** Jasper Lamers, Yanxia Zhang, Eva van Zelm, Cheuk Ka Leong, A. Jessica Meyer, Thijs de Zeeuw, Francel Verstappen, Mark Veen, Ayodeji O. Deolu-Ajayi, Charlotte M. M. Gommers, Christa Testerink

**Affiliations:** ^a^Laboratory of Plant Physiology, Plant Sciences Group, Wageningen University and Research, Wageningen 6708 PB, The Netherlands

**Keywords:** abscisic acid, salinity, root development, sodium stress, salt signaling

## Abstract

Soil salinity presents a dual challenge for plants, involving both osmotic and ionic stress. In response, plants deploy distinct yet interconnected mechanisms to cope with these facets of salinity stress. In this investigation, we observed a substantial overlap in the salt (NaCl)-induced transcriptional responses of *Arabidopsis* roots with those triggered by osmotic stress or the plant stress hormone abscisic acid (ABA), as anticipated. Notably, a specific cluster of genes responded uniquely to sodium (Na^+^) ions and are not regulated by the known monovalent cation sensing mechanism *MOCA1*. Surprisingly, expression of sodium-induced genes exhibited a negative correlation with the ABA response and preceded the activation of genes induced by the osmotic stress component of salt. Elevated exogenous ABA levels resulted in the complete abolition of sodium-induced responses. Consistently, the ABA insensitive *snrk2.2/2.3* double mutant displayed prolonged sodium-induced gene expression, coupled with increased root cell damage and root swelling under high salinity conditions. Moreover, ABA biosynthesis and signaling mutants were unable to redirect root growth to avoid high sodium concentrations and had increased sodium accumulation in the shoot. In summary, our findings unveil an unexpected and pivotal role for ABA signaling in mitigating cellular damage induced by salinity stress and modulating sodium-induced responses in plant roots.

## Significance

Soil salinization is an increasing agricultural problem world-wide. Yet, fundamental knowledge on how plants respond to salinity is still largely lacking. Through transcriptomics studies, we identified a Na^+^-specific cluster of up-regulated genes that showed an opposite response to Na^+^-containing salts compared to osmotic stress and that was surprisingly repressed by addition of abscisic acid (ABA). Next, we found that both sodium-specific gene expression and phenotypic responses of roots can only happen due to a delayed ABA response, induced by the osmotic component of the salinity stress. Our findings challenge the prevailing consensus that ABA consistently has a general promoting role in abiotic stress responses, providing insights for future studies on abiotic stress responses in plants.

Plants are bound to their location and must adequately respond to their environment to survive. This requires the translation of environmental cues to cellular signaling, which is achieved by various sensing mechanisms. Soil salinization is one of the major threats to agriculture affecting >1 billion hectares worldwide, including 20% of all irrigated farmlands (1).

High soil salinity lowers the water potential, which limits plant water content and causes osmotic stress, which is putatively sensed by mechanosensitive Ca^2+^ channels (2). In addition, toxic ions (sodium and chloride) cause ionic stress due to their accumulation in plants, inhibiting cellular processes (3, 4). Some plants, including mangrove trees (5) and quinoa (6), have evolved mechanisms to deal with extremely high salt concentrations, and are called halophyte species. On the other hand, the majority of plants, including most important crops, are highly salt sensitive (termed glycophyte species) (3). Interestingly, glycophytes can redirect root growth to specifically avoid high sodium concentrations in soil (7), a process named halotropism, which is not induced by an equimolar level of osmotic stress or by other monovalent cations, such as K^+^ or Li^+^ (7–9). This indicates the existence of a yet unexplored sodium-induced sensing mechanism in plants that allows plants to mount responses to this toxic ion that is increasingly present in natural and agricultural soils (1).

On the other hand, many responses to salt overlap with those induced by osmotic stresses, including drought. The plant hormone abscisic acid (ABA) is produced in response to many water-limiting abiotic stresses (such as drought, osmotic stress, salinity, cold, and frost) and has an important role in the regulation of germination, stomatal closure (10), and root development (11). ABA is sensed by binding to the *PYRABACTIN RESISTANCE1 (PYR)/PYR1-LIKE* family of receptors (12), which releases the inhibition by PROTEIN PHOSPHATASES TYPE 2C proteins (13) of the SUCROSE NON-FERMENTING 1-RELATED PROTEIN KINASE2 family [SnRK2; 2.2, 2.3 and 2.6 in *Arabidopsis* (14)]. SnRK2s activate downstream transcription factors (TFs) such as ABA INSENSITIVE 3 (ABI3), ABI4, ABI5 and ABA-RESPONSIVE ELEMENT-BINDING FACTOR/ABA RESPONSIVE ELEMENT-BINDING FACTOR 1 TFs (AREB/ABF; four homologs in *Arabidopsis*) (15, 16). In response to salt, ABA accumulation and ABA-induced gene expression peak at 3 to 6 h after salt application in *Arabidopsis* roots and decrease again after 24 h (17, 18), which is similar to the response of plants to water deficit (18).

Until recently it was widely accepted that the osmotic responses induced by salt precede those induced specifically by the ionic stress, which would occur only after sodium and chloride ions have accumulated to toxic levels (3). The discovery of the sodium-specific halotropism response (7, 9), and the rapid (<20 s) induction of Ca^2+^ waves migrating from root to shoot after root exposure to salt, but not osmotic stress (19, 20), have challenged this longstanding idea.

To understand how plant roots integrate the osmotic and ionic component in the early response to salt, we first sought to identify specific sodium-induced transcriptional responses in *Arabidopsis*. We performed an RNA sequencing experiment on roots of 8-d-old seedlings with two sodium treatments (NaCl and NaNO_3_), an ionic control (KCl), and an osmotic control (sorbitol) at 6 and 24 h. We found that genes that are specifically induced by sodium ions, are induced earlier than those general to all osmotic stresses and are negatively regulated by ABA. Further investigation of the relation between ABA and sodium-induced responses revealed that endogenous ABA signaling not only plays a general role in abiotic stress responses but is also required to specifically attenuate rapid sodium-induced responses in roots. Timing of ABA accumulation acts to limit sodium-induced cell damage and fine-tunes salt-induced changes in root growth and morphology.

## Results

### Salinity Triggers Sodium-Induced Gene Expression That Is Inhibited by ABA.

**Fig. 1.**
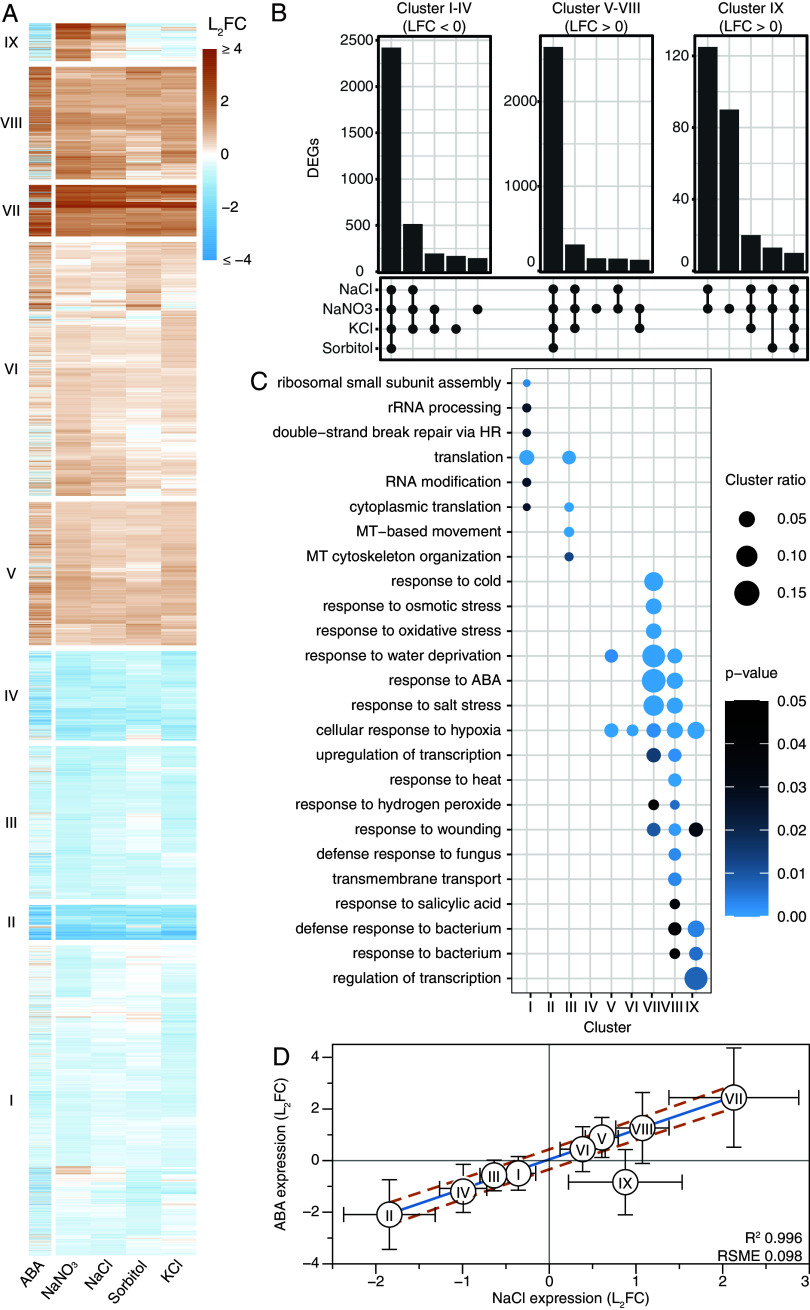
The *Arabidopsis* root transcriptome response to sodium ions negatively correlates with the response to ABA. Transcriptomics on 8-d-old *Arabidopsis* roots treated with 130 mM NaCl, 130 mM NaNO_3_, 130 mM KCl, 260 mM Sorbitol, or 25 µM ABA for 6 h. Transcripts were filtered for significance (FDR < 0.05) and expression (LFC > |0.5|) and were included when these conditions were met in at least 1 of the 4 treatments (NaCl, NaNO_3_, KCl, or sorbitol). This resulted in a selection of 8,431 DEGs. (*A*) Heatmap of the 8,431 DEGs. Treatments (columns) and transcripts (rows) were clustered with Euclidean distance mapping and Ward clustering. ABA treatment was not used for clustering and was added as a separate column. The heatmap was divided into nine clusters, which is indicated on the left side of the cluster. (Full data in Dataset S1). (*B*) Upset plots of shared DEGs (vs. control) per cluster pattern, up- (V-VIII) down- (I-IV) or sodium-induced (IX) regulation. The five most abundant interactions are shown here, see *SI Appendix*, Fig. S2*B* for full plots. (*C*) GOterm enrichment per cluster was analyzed with GOseq (21). GO terms containing less than 10 genes in total were excluded from the plot. The color indicates the Benjamini–Hochberg adjusted *P*-value. Size indicates the ratio of DEGs in the GO term to the total number of genes in the term. (Full data in Dataset S2). (*D*) Scatterplot of the mean L_2_FC in ABA and NaCl per cluster. The linear fit of cluster I-VIII is shown in blue, with a 99.99% prediction plot shown in red lines.

To identify sodium-induced transcriptional changes, 8-d-old *Arabidopsis* seedlings were subjected to mock (control), 130 mM ionic (NaCl, NaNO_3_, KCl), 260 mM osmotic (sorbitol) or 25 µM ABA treatments on solid ½ Murashige and Skoog (MS) medium. Roots were harvested after 6 and 24 h. The principal component analyses (PCA) of both timepoints show that the treatments caused more distinct expression patterns after 6 h compared to 24 h (*SI Appendix*, Fig. S1 *A* and *B*). Next, the ionic (NaCl, NaNO_3_, KCl) and osmotic (sorbitol) treatments were used to identify sodium-induced gene expression. 8,431 genes that were differentially expressed [false discovery rate (FDR) < 0.05, Log_2_ fold change (LFC) > |0.5|)] in at least one of the treatments compared to control [differentially expressed genes (DEGs)] were clustered to identify common expression patterns in response to the different treatments. Clustering was performed using Euclidean distance mapping and Ward clustering. As we aimed to dissect the sodium-induced transcriptional response from potential K^+^, Cl^−^, and osmotic transcriptional responses, the ABA treatment was not included in the selection of DEGs or the clustering itself and was added as a separate column after clustering. At 6 h, the genes were divided into nine clusters (Fig. 1*A*). Four clusters (I-IV) are predominantly downregulated by all treatments, while four others (V-VIII) are upregulated (Fig. 1*B* and full graphs in *SI Appendix*, Fig. S1*C*). On the contrary, genes in cluster IX show upregulation by sodium (NaCl, NaNO_3_), but not by sorbitol, KCl or ABA. A gene ontology (GO) term enrichment analysis showed that genes in the downregulated clusters (I-IV) are mostly related to translation, and those in the upregulated clusters (IV-VIII) enriched for terms related to abiotic stresses (Fig. 1*C*). Surprisingly, GO terms associated with (defense) response to bacterium and wounding, were strongly enriched in the sodium-induced cluster (IX) at 6 h. Consistent with the PCA plots, no strong sodium-induced response was identified at 24 h (*SI Appendix*, Fig. S4), which indicates that the distinct sodium-induced response is transient.

To identify putative upstream regulators, we studied the enrichment of TF binding sites in promoters of our DEGs for each gene cluster using PlantRegMap database (22). For 206 TFs we observed an enrichment of predicted binding sites (FDR < 0.05), of which 91 were enriched for multiple clusters (*SI Appendix*, Fig. S2*A*). Strikingly, there was a distinct separation of predicted TF binding sites for the general downregulated (I-IV) and upregulated clusters (V-VIII). For the sodium-induced cluster (IX), 47 TFs were enriched (FDR < 0.05) of which 42 were in the ARABIDOPSIS NAM, ATAF1/2, CUC2 (ANAC) TF family. We selected several *anac* mutants and investigated their sodium-specific inhibition of root gravitropism (23) and root growth as a proxy for defects in sodium-induced signaling, but did not find major differences compared to wild-type for these mutants (*SI Appendix*, Fig. S2*B*).

Next, we investigated other possible mechanisms of sodium-specificity. The mean NaCl response of genes in cluster I-VIII positively correlated with the response to ABA (R^2^ = 0.996, Fig. 1*D*), as expected. Interestingly, this correlation is not observed in the mean transcriptional response of cluster IX genes (Residual SD > 2.5 σ). Instead, the genes in this cluster are generally repressed by the ABA treatment. Based on our transcriptomics data, we thus hypothesize that an increase in ABA, caused by the osmotic component of NaCl stress, could potentially compromise the sodium-induced response to salinity (cluster IX).

### Sodium-Induced Gene Expression Peaks at Early Timepoints.

**Fig. 2.**
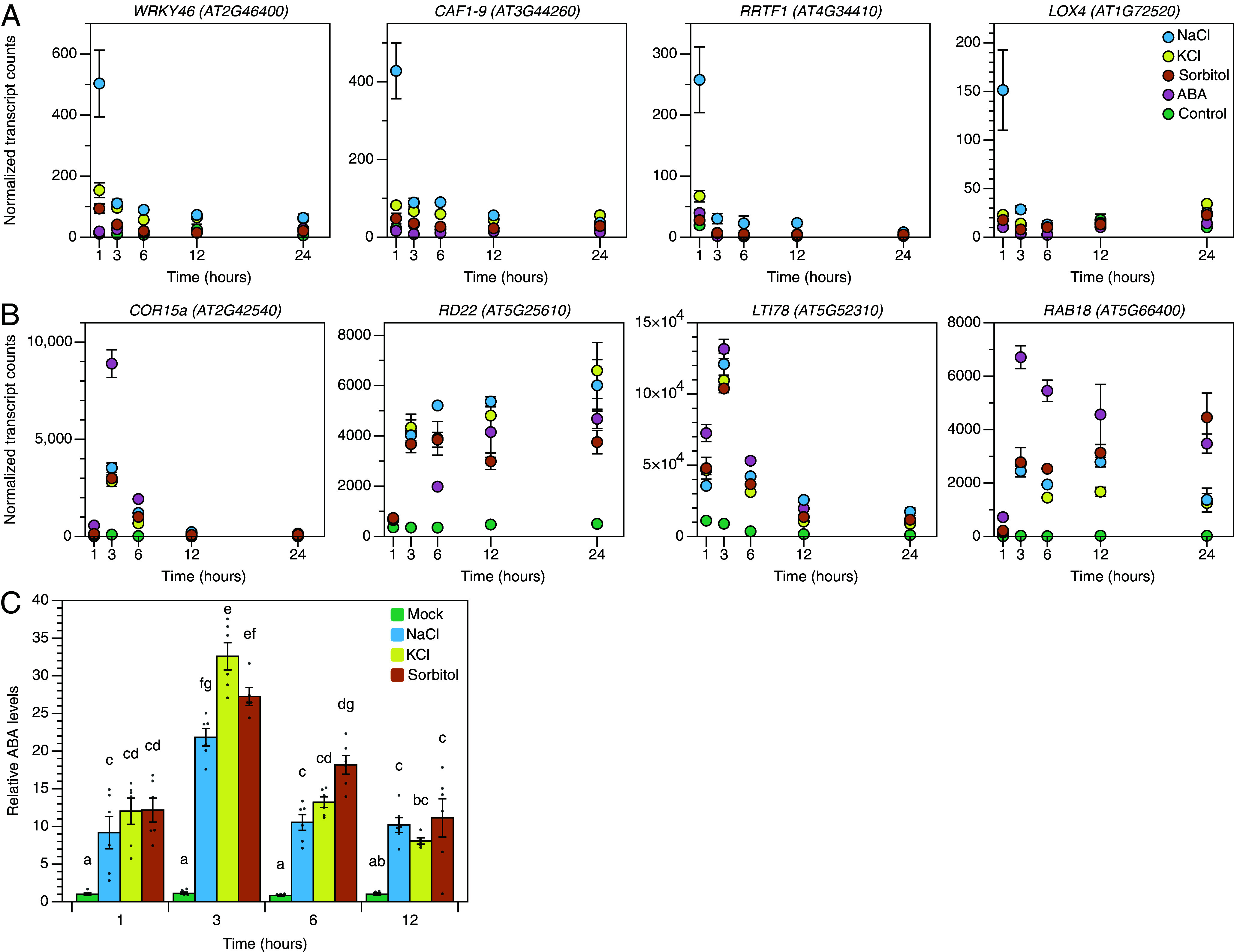
Sodium-induced gene expression precedes ABA responses. (*A* and *B*) Normalized transcript counts over time for (*A*) four highly expressed sodium-induced genes and (*B*) well-known ABA marker genes from RNA sequencing of 7-d-old seedlings after transfer to control, 125 mM NaCl, 125 mM KCl, or 250 mM sorbitol plates. Sodium-induced was defined as being significantly regulated at any timepoint by NaCl compared to all other treatments (control, KCl, and sorbitol). Dots represent the mean (n = 3) ±SE. Data was statistically analyzed using two-way ANOVA, followed by a Tukey post hoc test. (*C*) Normalized ABA levels compared to the mock treatment at 1 h over time in roots of 7-d-old seedlings after transfer to 125 mM NaCl, 125 mM KCl, or 250 mM sorbitol. Bars represent mean values (n = 5 to 6) ±SE and individual datapoints are shown as dots. Data was statistically analyzed using two-way ANOVA, followed by a Tukey post hoc test. Letters indicate significant differences (*P* < 0.05).

We hypothesized that the activation of sodium-induced gene expression and the induction of ABA signaling by high salinity could be temporarily separated. Due to the low abundance of Cluster IX transcripts (*SI Appendix*, Fig. S3), even in the NaCl treatment, few genes are suitable for detection with qPCR. Therefore, we performed a second RNA sequencing experiment on 7-d-old *Arabidopsis* roots that were harvested at 1, 3, 6, 12, or 24 h after transfer to control, 125 mM NaCl, 125 mM KCl, 250 mM sorbitol or 5 µM ABA treatments. For these experiments, we selected a concentration of 5 µM ABA, which approximates gene expression changes of ABA marker genes as did 125 mM NaCl (*SI Appendix*, Fig. S5). First, we selected genes of Cluster IX that were also sodium-induced at any timepoint in the second RNA sequencing dataset. This resulted in a set of 20 genes that were robustly sodium-induced (FDR < 0.05, Fig. 2*A* and *SI Appendix*, Fig. S7*A*), despite differences between experimental setups between RNA sequencing experiments and consistently low transcript counts for these genes (*SI Appendix*, Fig. S6*B*). In general, sodium-induced genes were induced within an hour after salt application, and were quickly reduced after 1 or 3 h, followed by a stable level of expression and a further decrease at 24 h (Fig. 2*A* and *SI Appendix*, Fig. S6*A*). Next, we investigated the temporal regulation of well-described ABA signaling marker genes in the same (time series) RNA sequencing dataset. ABA marker genes all peaked at 3 h after treatment application and stayed elevated for several hours (Fig. 2*B*). To investigate how these expression patterns relate to the synthesis of ABA in roots, we quantified ABA levels at 1, 3, 6, and 12 h after NaCl, KCl and sorbitol treatment. ABA levels significantly increased after 1 h, peaked at 3 h for all three stress treatments, and slowly declined afterward (Fig. 2*C*). Taken together, these results indicate that the early sodium-induced gene expression is most apparent 1 h after stress, and is later reduced, while ABA levels are still increasing.

### Sodium-Induced Gene Expression Is Enhanced in the *snrk2.2/2.3* Mutant and Is Independent of MOCA1 Function.

**Fig. 3.**
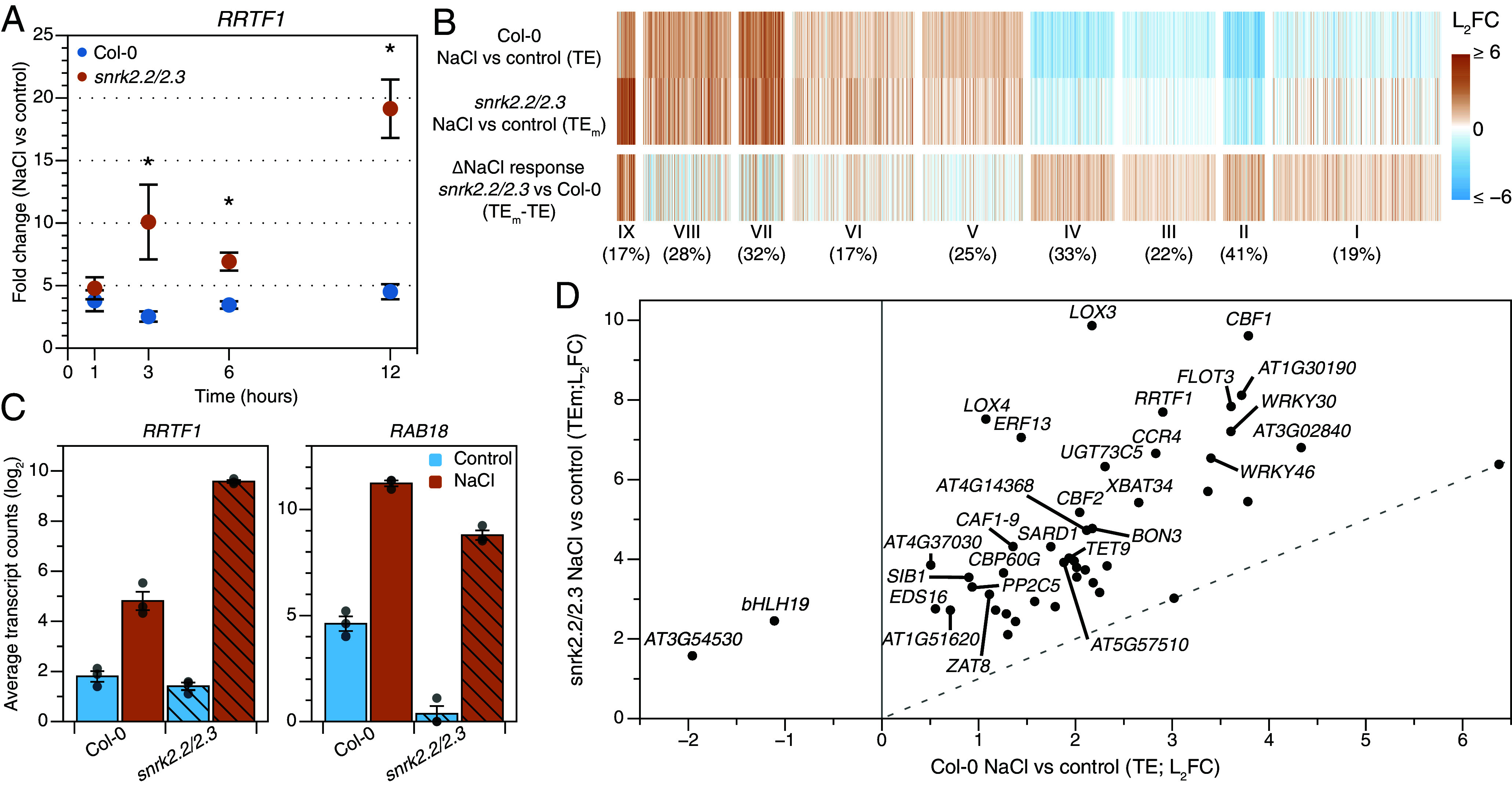
Endogenous ABA signaling is essential for the repression of sodium-induced gene expression. (*A*) Relative gene expression measured by Q-RT-PCR (Fold change NaCl vs. control) of *RRTF1* in the *snrk2.2/2.3* mutant and Col-0. 7-d-old seedlings were transferred to control or 125 mM NaCl medium and harvested after 1, 3, 6, and 12 h. Dots represent the mean (n = 3 to 4) ±SE and individual datapoints are shown as dots. Data was statistically analyzed using pairwise Welch tests between the genotypes per timepoint and corrected for multiple testing with Benjamini–Hochberg (BH). Asterisks indicate significant differences between genotypes (*P* < 0.05). (*B*) Heatmap of the 1,911 overlapping genes from RNA sequencing that were differentially regulated by NaCl in Col-0 and had a differential response in *snrk2.2/2.3* compared to Col-0 (FDR < 0.05). The clustering of the heatmap in Fig. 1*A* was used, but note that fewer genes were included due to different selection criteria. The numbers below the cluster number represent the percentage of genes overlapping genes between both RNA sequencing experiments. 1,161 genes were enhanced or suppressed in *snrk2.2/2.3*xNaCl IT, but not differentially expressed in Fig. 1 and therefore excluded from this visualization (See *SI Appendix*, Fig. S10*C* for the analysis including these genes). (Full data in Dataset S3). (*C*) Normalized counts of *RRTF1* and *RAB18* in Col-0 and *snrk2.2/2.3* at 3 h. Bars represent averages ±SE (n = 3). (*D*) Sodium induction of genes in cluster IX (Panel *B*) in Col-0 vs. *snrk2.2/2.3*. The name of genes with a L_2_FC difference >2 between Col-0 and *snrk2.2/2.3* are labeled. The dashed line indicates an equal regulation between Col-0 and *snrk2.2/2.3* (L_2_FC – 0).

From these two transcriptomics datasets, we identified *REDOX RESPONSIVE TF 1* (*RRTF1*) as a robustly RT-qPCR detectable sodium-induced marker gene, of which its NaCl-induced expression increases with the NaCl concentration applied (*SI Appendix*, Fig. S7). To investigate whether sodium-induced gene expression is affected by ABA signaling, we studied the temporal expression of *RRTF1* in roots of 7-d-old seedlings in the ABA-insensitive *snrk2.2/2.3* double mutant at 1, 3, 6, and 12 h after NaCl stress (Fig. 3*A* and *SI Appendix*, Fig. S8*A*). This sodium-induced marker gene was found to be significantly higher in NaCl-treated *snrk2.2/2.3* mutants compared to Columbia-0 (Col-0) from 3 h onward. Consistently, ABA biosynthesis mutants *aba2* and *aba3* (*SI Appendix*, Fig. S8 *B* and *C*) and the ABA insensitive *abi1-1* mutant (*SI Appendix*, Fig. S8*D*) also show enhanced *RRTF1* expression at 6 h after salt stress induction. Thus, endogenous ABA signaling can repress the sodium-induced marker gene RRTF1. As expected, the ABA signaling marker gene *RAB18* showed strongly reduced induction by salt in the *aba2*, *aba3*, *abi1-1,* and *snrk2.2/2.3* mutants compared to Col-0 and peaked at 3 h after stress induction in the wildtype (*SI Appendix*, Fig. S8 *A*–*D*) (17, 24).

To obtain a more comprehensive overview of the role of endogenous ABA signaling in sodium-induced gene expression during the response of roots to high salinity, we included *snrk2.2/2.3* at 1 and 3 h in our second RNA sequencing experiment. These are the consecutive timepoints at which the highest sodium-induced gene expression was detected (1 h; Fig. 2*A* and *SI Appendix*, Fig. S7*A*) and where *snrk2.2/2.3* mutant shows a large difference (3 h; Fig. 3*A*) with wild-type. First, we selected genes that had a 1) differential (FDR < 0.05) NaCl response (NaCl vs. control) in *snrk2.2/2.3* compared to Col-0 (also termed Interaction Term; *SI Appendix*, Fig. S9*A*) and 2) were differentially regulated by NaCl in Col-0. We identified 180 and 3,072 genes that met both criteria at 1 and 3 h after stress induction, respectively (*SI Appendix*, Fig. S9*B*), which aligns well with the relatively slow offset of ABA response (Figs. 2 *B* and *C* and 3*A*). Next, we analyzed the 3-h timepoint in more detail by grouping these selected genes using the clusters as identified in our first transcriptomics experiment (Fig. 1 and *SI Appendix*, Fig. S9*C*). While in this case only DEGs were included that were both significantly regulated by NaCl and differentially responding in *snrk2.2/2.3* compared to wildtype at 3 h (in total 3,072 genes), the treatment effect (NaCl vs. control) in Col-0 showed a highly similar pattern as observed in Fig. 1*A*, in which cluster I-IV and cluster V-IX were down- and upregulated, respectively (Fig. 3*B*). The genes in clusters I-VIII generally showed reduced salt-induced changes in expression in *snrk2.2/2.3* compared to wildtype (Fig. 3 *C* and *D* and *SI Appendix*, Fig. S9 *D* and *E*). Strikingly, genes in the sodium-induced cluster IX, on the other hand, had a strongly enhanced response in *snrk2.2/2.3*. In other words, these salt-upregulated genes were even higher upregulated by salt in *snrk2.2/3.3* (Fig. 3*D* and *SI Appendix*, Fig. S9 *D* and *E*). This confirms that endogenous ABA robustly represses sodium-induced genes.

To investigate whether the previously reported monovalent cation sensor MOCA1 would be involved in the observed sodium-induced responses, we had included the *moca1* mutant (25) in our RNA sequencing experiment as well. Only 52 genes showed a differential response to NaCl in *moca1* vs. wildtype (FDR < 0.05; *SI Appendix*, Fig. S10*A*). No difference was found in NaCl-induced *RRTF1* expression in this mutant compared to wildtype (*SI Appendix*, Fig. S10*B*). Interestingly, *moca1* mutant seedlings are also not impaired in halotropism. Instead, the *moca1* mutant shows a hypersensitive root halotropic response, similar to *salt-overly-sensitive* mutants (7) (*SI Appendix*, Fig. S10*C*). In conclusion, the sodium-induced responses identified in this paper are not downstream of the monovalent cation sensor MOCA1 and are likely induced by an independent sodium-perception mechanism.

### Endogenous ABA Is Required to Mitigate Sodium Stress.

**Fig. 4.**
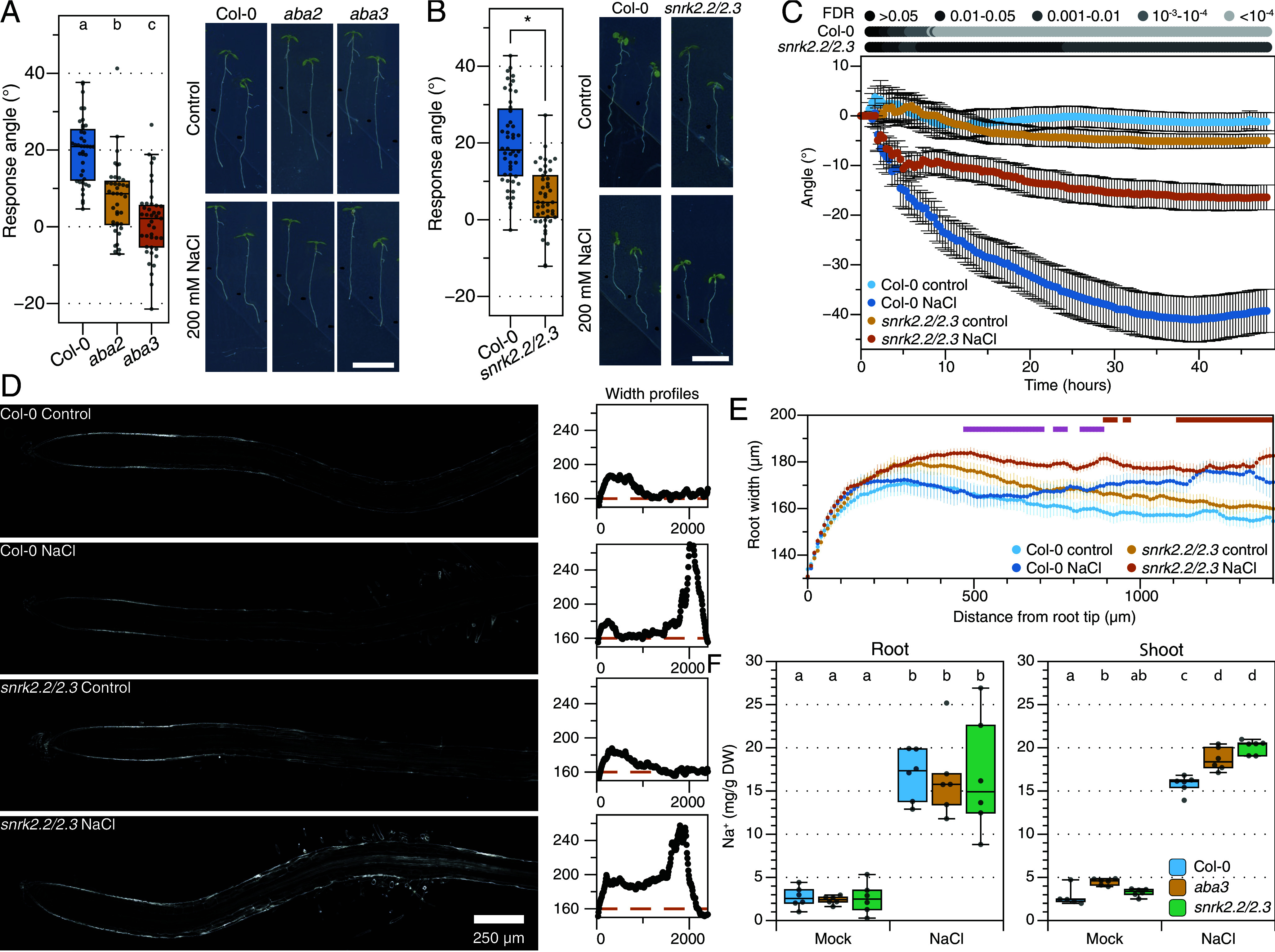
ABA signaling is essential for an adequate salt-specific response of roots. (*A* and *B*) Quantification of the halotropism response of ABA biosynthesis (*A*) and signaling (*B*) mutants at 48 h after the introduction of the NaCl gradient. The response angle shows the difference between control and NaCl conditions for the respective mutant. n = 35 to 41 and individual datapoints are shown as dots. Letters and asterisk indicate significant differences (*P* < 0.05), calculated by nonparametrical Dunn’s test and corrected for multiple testing using BH procedures. The data shown in panel *B* is a selection of a larger experiment of which the data, statistics, and representative images are entirely shown in *SI Appendix*, Fig. S11 *B* and *C*. (*C*) Halotropism time-series with 5-d-old seedlings of *snrk2.2/2.2* double mutants and Col-0 from the moment that the sodium gradient is created. Seedlings were imaged every 20 min for 48 h. Dots represent mean values (n = 20) ±SE. Pairwise Welch tests were performed for NaCl-treated samples and the control condition of the same genotype and corrected for multiple testing with BH. Corrected *P*-values (NaCl vs. control) are shown in gray-values above the graph. (*D*) Confocal images of calcofluor-white stained Col-0 and *snrk2.2/2.3* roots at 24 h after NaCl treatment. The diameter profiles of the depicted roots are shown on the *Left*, with the distance from the root tip (µm) on the *x*-axis and the diameter (µm) on the *y*-axis. The orange dotted line indicates average root diameter under control conditions. (*E*) Quantification of root diameter ratio along the root axis (length 0 = root tip) for Col-0 and *snrk2.2/2.3* as shown in Panel *C*. The orange bars above the graph indicate significant differences (FDR < 0.05) between NaCl-treated *snrk2.2/2.3* roots and control as analyzed using multiple Welch tests followed by BH correction for multiple testing. The pink bars indicate significant differences between salt-treated Col-0 and *snrk2.2/2.3* roots. Col-0 roots were not significantly different between NaCl and control. (*F*) Sodium content in roots and shoots of Col-0, *aba3* and *snrk2.2/2.3* at 6 h after a 125 mM NaCl treatment. Letters indicate significant differences, analyzed by two-way ANOVA, followed by a Tukey post hoc test, *P* < 0.05.

As endogenous ABA signaling repressed sodium-induced gene expression, we investigated whether it also affects other root responses to sodium stress. First, we studied halotropism in various mutants with defects in ABA biosynthesis (*aba2* and *aba3*) or signaling: *abi1-1*, the *pyr1/pyl1/2/4* ABA receptor mutant (referred to as *abaQ*), the *snrk2.2/2.3* protein kinase mutant and mutants for downstream positive regulators of ABA signaling: *abi3-8, abi4-3*, *abi5-7* and *areb1/areb2/abf3/abf1-1* (referred to as *arebQ*; *SI Appendix*, Fig. S11*A*). The halotropism response was significantly reduced in biosynthesis mutants (Fig. 4*A*) and signaling mutants *abi1-1*, *abaQ,* and *snrk2.2/2.3*, showing that core ABA signaling is essential for root directional avoidance of sodium (Fig. 4*B* and *SI Appendix*, Fig. S11 *B–E*). All investigated mutants for downstream ABA signaling components, on the other hand, showed a wildtype halotropism response (*SI Appendix*, Fig. S11 *B* and *C*). This could indicate genetic redundancy in downstream ABA signaling or the involvement of other downstream targets that were not tested here. Next, we also studied a mutant of our selected sodium-specific marker gene *RRTF1*, but *rrtf1* mutants showed a wildtype halotropism response (*SI Appendix*, Fig. S4*B*), indicating that it is not required for halotropism.

To study the temporal characteristics of halotropism in the *snrk2.2/2.3* mutant, 5-d-old plants were placed in a timelapse imaging setup for 48 h after the introduction of the NaCl gradient (Fig. 4*C*). Both Col-0 and snrk2.2/2.3 seedlings quickly changed the growth direction of their main root to avoid high sodium concentrations and were significantly different (FDR < 0.05) from control conditions after 2:40 h and 3:00 h, respectively. However, the halotropism response stagnated in the *snrk2.2/2.3* mutant after approximately 5 h. Even though roots of the *snrk2.2/2.3* mutant grew slower than wildtype roots (*SI Appendix*, Fig. S12), plotting root growth vs angle shows that this could not explain the difference in root angle response between the genotypes. (*SI Appendix*, Fig. S12). Taken together, these results show that the initial sodium-avoidance response is independent of ABA, but is required to drive sodium avoidance at later timepoints.

Previous work reported NaCl-induced cell swelling in the early elongation zone, which is not observed by a comparable osmotic treatment (26) (and confirmed here in *SI Appendix*, Fig. S13*D*). Hence, we asked whether endogenous ABA signaling also affects cell morphology during salt stress, which we investigated by measuring root diameter at 24 h after salt stress induction (*SI Appendix*, Fig. S13 *A*–*C*). The initial root swelling (1,400 to 2,300 µm zone) was comparable between Col-0 and *snrk2.2/2.3* (*SI Appendix*, Fig. S13*E*). However, salt-treated *snrk2.2/2.3* roots showed significantly more cell swelling compared to control grown seedlings in the region closer to the root tip (900 to 1,500 µm zone) and salt-treated *snrk2.2/2.3* roots were significantly wider than salt-treated Col-0 roots (at 500 to 900 µm from the tip; Fig. 4 *C* and *D*), while salt-treated Col-0 roots were not significantly different in diameter compared to control treated roots. Similar results were observed for ABA biosynthesis mutants *aba2*, *aba3,* and the ABA signaling mutant *abi1-1* (*SI Appendix*, Fig. S13 *F–H*).

Next, we wondered whether ABA is important for the regulation of ion levels in seedlings. Therefore, we investigated Na^+^, Cl^−^, and K^+^ levels in the *aba3* biosynthesis and *snrk2.2/2.3* signaling mutants at 6 h after the induction of salt stress. Strikingly, no differences in Na^+^ or Cl^−^-content were found in the root, but shoots of *aba3* and *snrk2.2/2.3* mutants showed increased sodium and chloride levels (Fig. 4*F* and *SI Appendix*, Fig. S14 *A* and *B*). K^+^ levels were lower in roots of NaCl-stressed *aba3* mutants and higher in shoots of *aba3* and *snrk2.2/2.3* mutants under both control and NaCl conditions. *rrtf1* mutants showed wildtype ion contents in roots and shoots in control and salt conditions.

Taken together, this shows that endogenous ABA signaling is important for roots to recover from sodium-induced stress on the transcriptional and cellular levels. As an independent line of evidence, we found that *rbohc* and *rbohf* mutants have strongly reduced expression of ABA signaling marker genes and phenocopy *snrk2.2/2.3* for sodium-induced responses (*SI Appendix*, Fig. S15).

### ABA Pretreatment Inhibits Sodium-Induced Responses.

**Fig. 5.**
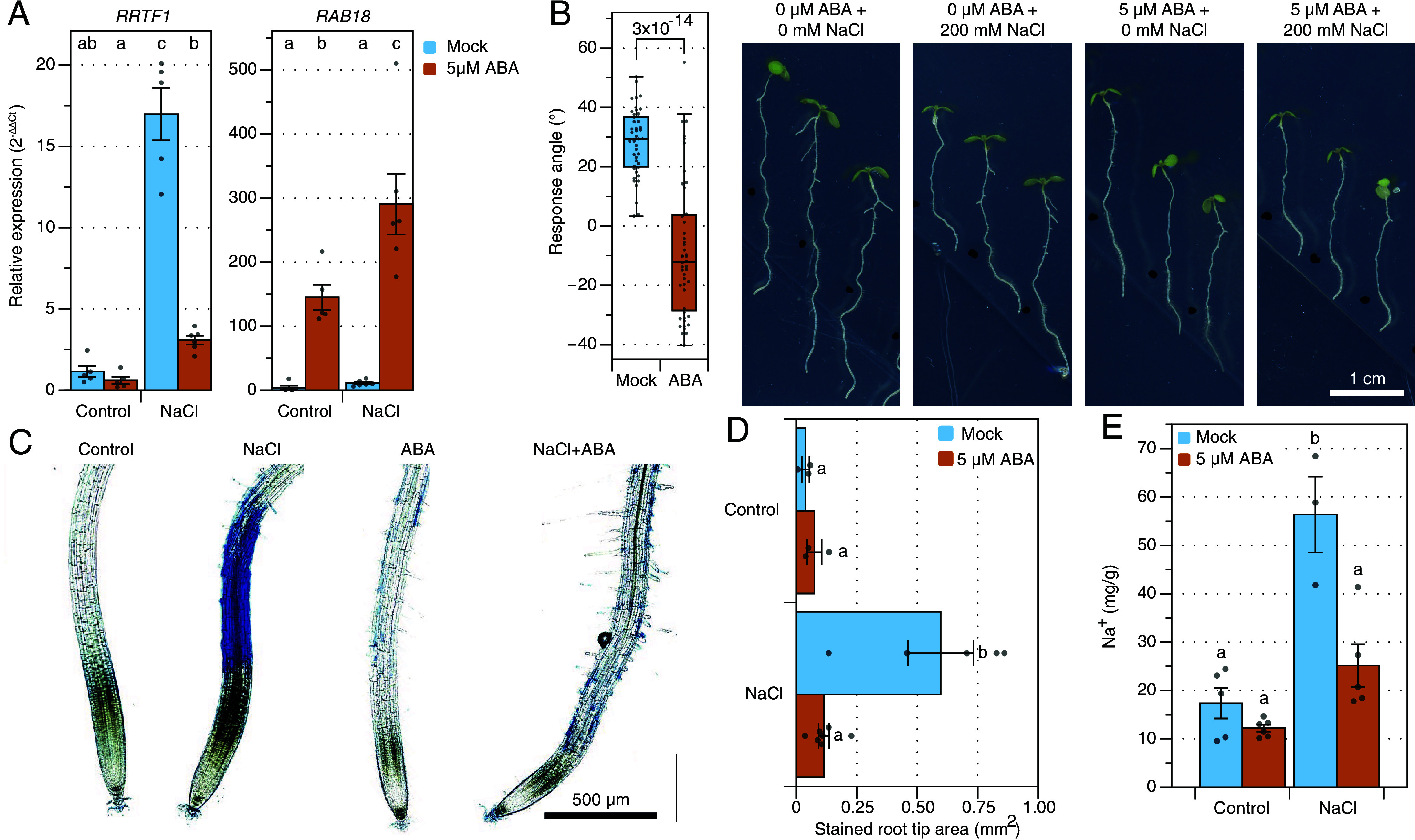
ABA represses sodium-induced responses. (*A*) Relative gene expression of a sodium-induced transcript (*RRTF1*) and an ABA signaling marker (*RAB18*) in roots of 7-d-old seedlings after transfer to control or 125 mM NaCl medium for 1 h and with (orange) or without (blue) 5 µM ABA pretreatment for 12 h. Expression was normalized to the control condition without ABA. Bars represent mean values (n = 5 to 6) ±SE and individual datapoints are shown as dots. Statistical analysis was performed using nonparametrical Dunn’s test on the log_2_ transformed data and corrected for multiple testing using BH procedures. Letters indicate statistical groups. (*B*) Quantification and representative images of the halotropism response at 48 h after introduction of the gradient in the presence or absence of an ABA pretreatment. The response angle shows the difference between control and NaCl conditions for the respective ABA treatment. The relative growth shows the ratio between NaCl and control conditions for the respective ABA. Statistics were performed using pairwise *t* tests. (*C*) Representative images of Evans Blue staining for plasma membrane damage at 48 h after the introduction of the gradient of the halotropism assay. (*D*) The affected area of the assay (*C*) was quantified using a script (*SI Appendix*, Fig. S15). Bars represent mean values (n = 5) ±SE and individual datapoints are shown as dots. Letters indicate significant differences, analyzed by one-way ANOVA, followed by a Tukey post hoc test, *P* < 0.05. The midplane (1 stack) of the calcofluor-white cell wall staining is shown in white. White arrowheads indicate the start of the elongation zone based on the cortical cell length. (*E*) Sodium contents in roots of 7-d-old seedlings after transfer to control or 125 mM NaCl medium for 6 h and with (red) or without (blue) 5 µM ABA pretreatment for 12 h. Bars represent mean values (n = 3 to 6) ±SE and individual datapoints are shown as dots.

As we observed that sodium-induced responses are enhanced in the ABA signaling mutant *snrk2.2/2.3*, we questioned whether the exogenous application of ABA can also suppress sodium-induced responses. First, we quantified expression of the robustly quantifiable sodium-induced marker *RRTF1* and the ABA signaling marker gene *RAB18* after a combined treatment of ABA and NaCl. To maximize ABA signaling at the start of the NaCl application (Fig. 2*B*), seedlings were transferred to mock or 5 µM ABA treatment plates 12 h prior to the NaCl or control treatment with the corresponding ABA concentration. Gene expression of *RRTF1* was significantly (*P* < 0.05) upregulated after 1 h of NaCl stress, while the ABA pretreatment significantly reduced NaCl-induced expression of *RRTF1* to control levels (Fig. 5*A*). As expected, the ABA signaling marker gene *RAB18* was not induced after 1 h of salt stress, but was upregulated by ABA and the combination of ABA and NaCl. Taken together, these results confirm that the strong induction of the sodium-induced marker gene *RRTF1* at 1 h after salt treatment is facilitated by the low ABA levels at this stage (Fig. 2*C*). Next, we asked whether ABA would also repress the sodium-specific halotropism response of roots. To test this, 4.5-d-old seedlings were transferred to fresh plates with or without 5 µM ABA for 12 h prior to the introduction of the gradient. While roots avoided the sodium-gradient in the absence of ABA, the ABA-treated plants did not show a net sodium-avoidance at 24 h (*SI Appendix*, Fig. S16) and were even growing toward sodium at 48 h after the introduction of the gradient (Fig. 5*B* and *SI Appendix*, Fig. S16). Taken together this indicates that ABA inhibits sodium-induced root responses.

Next, intrigued by enrichment of the sodium-induced gene cluster IX for genes involved in wounding-related processes (Fig. 1*D*), we investigated whether salinity stress causes wounding to root cells. This was assessed using Evans Blue staining (27) after a halotropism assay in the presence or absence of an ABA pretreatment. No damage was detected in the control group, but the NaCl-treated roots were severely damaged in the elongation zone (Fig. 5*C*) and ABA reduced the NaCl-induced damage (Fig. 5 *C* and *D*). As the ABA pretreatment abolishes all investigated sodium-induced responses, we hypothesized that ABA lowers Na^+^ levels. Therefore, we quantified sodium content in 7-d-old roots at 6 h after a homogeneous control or 125 mM NaCl treatment, in the presence or absence of a 5 µM ABA pretreatment. ABA pretreatment strongly reduced the sodium content in the root (Fig. 5*E*). In conclusion, ABA lowers root Na^+^ content and consistently abolishes sodium-induced responses, including gene expression, halotropism, and cell damage in the elongation zone of roots exposed to high salinity.

### RRTF1 Is Expressed in the Elongation Zone and Is Independent of JA Signaling.

**Fig. 6.**
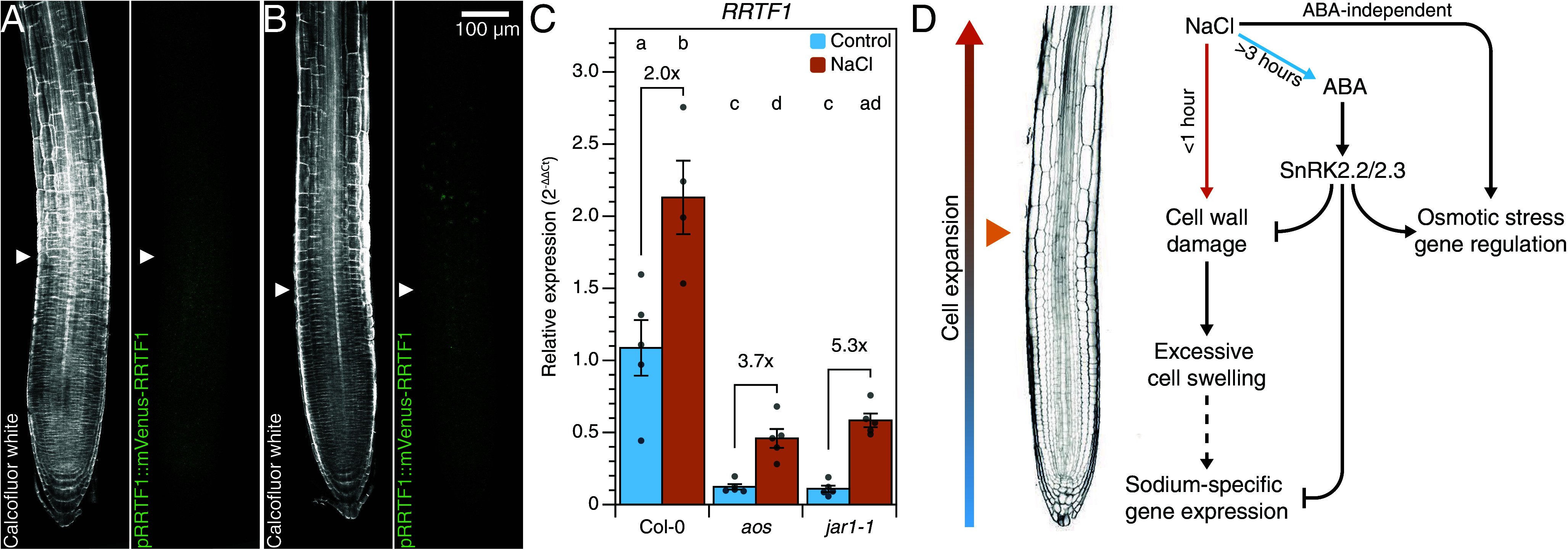
RRTF1, a sodium-induced marker gene, is expressed in the elongation zone during salt stress independent of JA signaling. (*A* and *B*) Maximum projection confocal images of roots expressing *pRRTF1::RRTF1-Venus* at 2 h after control (*A*) or 125 mM NaCl treatment (*B*). (*C*) qPCR analysis of sodium-induced *RRTF1* transcripts in roots of 7-d-old JA biosynthesis mutants and Col-0 control. Seedlings were treated with control or 125 mM NaCl for 1 h. Bars represent mean values ±SE, n = 5 and individual datapoints are shown as dots. Letters indicate significant differences by two-way ANOVA followed by a Tukey post hoc (*P* < 0.05). (*D*) A model on how acute sodium stress results in cell wall damage, which leads to cell damage in expanding cells in the elongation zone. There is a compelling correlation between damage and sodium-induced gene expression (<1 h). ABA accumulation and signaling is activated at later timepoints (>3 h), which inhibits cell damage and sodium-induced gene expression in a SnRK2.2/2.3-dependent manner. The orange arrow indicates the approximate location of RRTF1 expression, which functions as a marker for sodium-induced gene expression. Solid lines indicate direct evidence. The dashed lines are indirectly supported by our results and literature (see main text).

As our sodium-induced responses are not induced by previously described sensors (like *MOCA1*), we studied the potential mechanism that induces sodium-induced gene expression. First, we studied the localization of *p RRTF1:RRTF1-Venus* (28) at 1 to 3 h after salt stress. Interestingly, RRTF1-Venus accumulated in the elongation zone (600 to 850 µm from the root tip) in response to salt stress after 2 h (Fig. 6 *A* and *B* and *SI Appendix*, Fig. S17), which spatially overlaps with the NaCl-induced cell damage (Fig. 5*C*). This aligns well with the strongly enhanced NaCl-induced *RRTF1* expression in cell wall integrity mutants (29), which also show enhanced cell damage (*SI Appendix*, Fig. S14). Furthermore, this shows that *RRTF1* is expressed in few cells, which aligns well with the low transcript counts in the RNA sequencing dataset as described above (Fig. 1 and *SI Appendix*, Fig. S3). As *RRTF1* is known to be quickly induced by JA during mechanical damage (30, 31), we investigated whether its NaCl-induced expression is JA dependent. Even though control levels of *RRTF1* were significantly lower in mutants with reduced biologically active JA (*aos* and *jar1-1*), expression was still induced by NaCl (Fig. 6*C*). Finally, *aos* and *jar1-1* mutants showed wildtype root halotropic responses (*SI Appendix*, Fig. S18), all together indicating that early sodium-induced responses are JA-independent.

## Discussion

In this study, we report that in response to high salt, roots exhibit quick sodium-induced transcriptional responses (1-h poststress induction), which are repressed by ABA, which naturally accumulates in response to salinity at later timepoints. Modifying this natural course of action by means of an ABA pretreatment abolishes sodium-induced responses altogether (Fig. 5). Moreover, genetic inhibition of ABA signaling during salt treatment leads to 1) increased and prolonged sodium-induced gene expression (Fig. 3), 2) increased root cell swelling (Fig. 4 *C* and *D*), 3) increased shoot sodium and chloride levels (Fig. 4*F*) and 4) reduced negative root halotropism (Fig. 4*E*). Our data thus reveal an essential role for ABA in mitigating the effects of sodium stress.

We show that sodium-induced gene expression is quickly induced by salt stress but is restored to control levels even in the continuing presence of salt stress. This restoration depends on ABA signaling. While this dynamic regulation of sodium-induced gene expression seems surprising, it already has been reported that salt-stressed roots go through various phases of root growth during early salt stress. Namely, after exposure to NaCl, roots go through a stop (0 to 5 h), quiescent (5 to 9 h), recovery (9 to 12 h) and homeostasis (>12 h) growth phase (17, 29). Our timeseries show that sodium-induced gene expression is high at the early timepoints after stress induction (Figs. 2 and 3*A*), which corresponds with the stop phase. Later, the osmotic stress-induced ABA biosynthesis and signaling repress sodium-induced gene expression, which aligns with the timing of growth recovery. Therefore, we hypothesize that the acute sodium-stress induces sodium-induced gene expression and abortion of growth, and this sodium stress is alleviated by ABA signaling. This is further supported by the fact that chemical inhibition of ABA biosynthesis during salt stress inhibits growth recovery after the quiescent phase (17). Notably, *rbohc* and *rbohf*, but not *rbohd* mutants, also show reduced ABA marker gene (*RAB18*) expression under control and salt treatment, and enhanced sodium-induced gene expression and reduced halotropism. These mutants thus phenocopy ABA biosynthesis and signaling mutants with respect to sodium-induced responses of the root. This aligns well with existing literature which shows that responses to ABA (root growth and stomatal closure) are reduced in *rbohf*, but not in *rbohd* (32) mutants.

Currently, the function of identified sodium-induced genes remains ambiguous. The *rrtf1* mutant shows a wildtype response for the investigated sodium-specific responses (*SI Appendix*, Figs. S11*F* and S14), but sodium-specific genes are not limited to this gene and/or there might be gene redundancy. The identification of upstream regulatory elements might shed light on their regulation and function, but our transcription binding site enrichment approach did not yield promising candidates (*SI Appendix*, Fig. S2). Thus, we hypothesize that sodium-induced gene expression is not per se beneficial for the plant but is an output of the acute sodium stress. More importantly, we also show that an ABA pretreatment abolishes NaCl-induced cell damage and lowers sodium concentrations in the root, and ABA biosynthesis and signaling mutants show consistent opposite phenotypes. Thus, the ABA-mediated mitigation of acute sodium-stress has clear advantages for the plant. Another clear advantage is that ABA is required for normal halotropism (33), as ABA signaling mutants show strongly reduced halotropism (Fig. 4). This seems to contradict the reduced halotropism after the ABA pretreatment (Fig. 5), but as redirection of root growth depends on an asymmetry in cell elongation (33, 34), we hypothesize that disturbing this asymmetry either by genetically blocking or treating with ABA homogeneously, hampers the halotropic response. Interestingly, the *snrk2.2/2.3* halotropism timeseries data suggest that *snrk2.2/2.3* mutants can initially redirect root growth to avoid high salt concentrations like wildtype, but the response stagnates after 5 h (Fig. 4*B*). This aligns well with the halotropism phenotype after the ABA pretreatment, in which roots initially seem to bend (Fig. 5*B*), but no net root avoidance is measured after 24 h. Thus, while ABA is not essential for the initial redirection of root growth, it could be required for the maintenance of the sodium avoiding halotropic response at later timepoints.

To get a better understanding of sodium-induced regulatory mechanisms, we aimed to disentangle the sodium-induced, general monovalent cation, and osmotic responses to salt stress. We expected to identify monovalent-cation specific transcriptional responses, as salt stress specifically causes a rapid (<20 s) increase in intracellular Ca^2+^ which migrates in waves throughout the plant. These waves cause transcriptional changes in the shoot, which is not directly in contact with the saline soil (19). Recently, it was described that these Ca^2+^ waves are not sodium-induced, but are induced by other monovalent cations (Li^+^ and K^+^) as well, and are sensed by negatively charged sphingolipids [biosynthesized by MOCA1 (25)], which in turn activate yet unknown Ca^2+^ channels (25). In our transcriptome analysis, we were able to distinguish sodium-induced gene expression from a general osmotic stress response induced by both KCl and osmotic stress, as well as ABA treatment. However, no evident generic monovalent cation stress pattern was identified, which aligns well with the almost wildtype-like salt stress transcriptome of the monovalent cation sensor mutant *moca1*. In accordance, we found MOCA1 not to be required for the sodium-induced halotropism response (*SI Appendix*, Fig. S10). This suggests that monovalent cation-induced Ca^2+^ waves do not affect early sodium-induced responses in the roots in our conditions, leaving open the question of the molecular nature of sodium perception in plants.

Interestingly, the elongation zone is especially important for sodium-specific responses. Previously, it was already shown that the sodium-specific halotropic response is caused by differential cell elongation in this root zone (33, 34). We now showed that salt stress induces both cell damage and *RRTF1* expression in this root zone. Cells in this region undergo rapid elongation, which exhorts tension on the cell wall (35, 36). Sodium, however, affects cell wall integrity by disrupting pectin crosslinks, which reduces the capacity to resist turgor pressure and leads to salt-induced cell swelling (26). This effect is clearly visible upon genetic inhibition of cell wall integrity, like in the Feronia (Fer) mutant, which shows excessive salt-induced cell swelling (and even bursting) during salt stress (26), as well as strongly enhanced sodium-induced gene expression (29) and cell damage (*SI Appendix*, Fig. S19). Consistently, ABA biosynthesis and signaling mutants also exhibit increased cell swelling and enhanced sodium-induced gene expression in response to salt. The ABA pretreatment prevents both cell damage and sodium-induced gene expression, in alignment with previous reports in which NaCl-induced cell swelling is strongly enhanced when ABA biosynthesis is chemically inhibited (17). Concluding, ABA insensitivity weakly phenocopies the *fer* mutant phenotype. As cell elongation is driven by turgor pressure and the direction of elongation by the properties of the cell wall (35, 36), we propose that the NaCl-induced radial cell swelling in *snrk2.2/2.3* root cells is caused by damaged (or altered) cell wall properties. This line of reasoning is summarized in model in which acute sodium-stress leads to weakened cell walls, which induces cell damage responses and sodium-induced gene expression in the elongation zone (Fig. 6*D*). The osmotic component of NaCl stress leads to the accumulation of ABA signaling, which reverts the cell wall damage and the sodium-induced gene expression.

To conclude, we have shown that when plant roots are exposed to salinity, early sodium-induced gene expression is repressed by the slower osmotic stress-induced ABA accumulation and signaling. By enhancing (ABA treatment) or repressing (ABA-insensitive mutants) ABA signaling, we were able to show that the interaction between the stress components is required to mount an adequate response to salt while maintaining cell integrity. Our work gives insights in the tight temporal and spatial responses of a complex stress. We argue that salinity stress, like many abiotic stresses, triggers different response pathways, which are separated in time and space. The existence of a clearly specific response opens doors to investigate sodium sensing mechanisms and targets for salt stress resilience.

## Materials and Methods

### Plant Materials, Growth Conditions, and Stress Assays.

*Arabidopsis thaliana* plants of ecotype Col-0 were used in all experiments. Previously described lines were used here *aos* (37), *jar1-1* (38), *rbohc* (SALK_071801) (39), *rbohd-1* (SALK_070610C) (40), *rbohd-2* (SALK_120299C) (41), *rbohd-3* (42)*, rbohf-1* (SALK_059888) (43), *rbohf-2* (SALK_034674) (44), *aba2* (EMS) (45), *aba*3 (EMS) (45)*, abi1-1* (46), *snrk2.2/2.3* (GABI_807G04/ SALK_107315) (14), *abi3-8* (47), *abi4-3* (47), *abi5-7* (47), *abaQ* (*pyr1-1*/*pyl1-1*/*pyl2-1*/*pyl4-1;* Point/Salk_054640/CSHL_GT2864_1/Sail_517_C08) (12), arebQ (48), (*areb1*/*areb2*/*abf3*/*abf1-1;* SALK_002984/SALK_069523/SALK_096965/ SALK_132819), *pRRTF1:RRTF1-VENUS* (Named; *ERF109pro:ERF109-Venus*) (28), *moca* (25), *anac013-1D* (SALK_096150C) (49), *anac016-1* (SALK_001597C) (50), *anac037-1* (SALK_022534) (51), *anac056-1* (SM_3.28017) (52), *anac057-1* (SALK_086132), *anac092-1* (SALK_090154C) (53), *anac096-1* (SALK_078797C) (54).

Seeds were wet sterilized by 30% commercial bleach and 0.2% (v/v) triton-X 100 for 10 min and washed five times with sterile milliQ. Seeds were sown on ½ MS medium including vitamins (Duchefa) containing 0.1% 2-Morpholinoethanesulfonic acid monohydrate (MES) buffer (Duchefa) and 1% Daishin agar (Duchefa) and pH was adjusted to 5.8 with KOH. Seeds were stratified at 4 °C in the dark for 2 d. Plants were grown in a rack at an angle of 90° at 22 °C and long day photoperiod (16 h light, 120 µM/m^2^/s).

### Pharmacological Treatments and Root Harvesting.

Agar plates were covered with 1.5 × 10.5 cm 50 µM nylon mesh strips (Sefar BV) before sowing to facilitate seedling transfer to treatment plates. Plants were grown for 7 d [6.5 for the 12-h ABA (5 mM stock in ethanol) pretreatment] under the conditions described above and transferred to treatment plates. Roots were dissected with a sharp blade and flash frozen in liquid nitrogen. For RNA extraction and ion measurement, approximately 60 roots were pooled in one sample. This protocol was also used for the second RNA sequencing experiment (Figs. 2 and 3, with the only exception that 0.8% Plant agar (Duchefa) was used instead of Daishin agar.

For the first RNA sequencing experiment (Fig. 1), stress induction was performed as described above with minor changes. Instead of small mesh strips, the whole agar plate was covered by a 10.5 × 10.5 cm 50 µM nylon mesh. Plants were grown for 8 d at a 70° angle before transfer to 130 mM NaCl (Duchefa), 130 mM NaNO_3_ (Merck), 130 mM KCl (Merck), 260 mM Sorbitol (Duchefa), 30 mM LiCl (Merck), 25 µM ABA (Sigma, in ethanol) or control medium without supplements. Roots were harvested after 6 and 24 h. Approximately 40 roots from one plate were pooled as one biological replicate.

### RNA Isolation and qPCR.

Samples were homogenized using two stainless steel beads and Mixer Mill MM400 (Retsch), after which RNA was isolated with a Total RNA Isolation kit (NZYTech) (55). RNA quality was quantified using the Nanodrop™ One, followed by RQ1 DNase treatment (Promega) with 2,000 ng RNA. 1,000 ng was used for complementary DNA (cDNA) synthesis using iScript reverse transcriptase (Bio-Rad). cDNA was diluted to 10 ng/µL before quantification by RT-qPCR (25 ng cDNA per sample), using SYBR Green blue mix lo-rox (Sopachem) with a CFX96 real time system (Bio-Rad). Relative expression was calculated with the ΔΔCt method, using *MON1* (AT2G28390) as housekeeping gene. Primers used for RT-qPCR are listed in Dataset S6. Samples for RNA sequencing were isolated using the RNeasy Kit (Qiagen). For the first RNA sequencing in combination with Tripure (Roche) as previously published (55).

### Library Preparation, Sequencing, and Data Processing.

Total RNA was used for RNA library preparation suitable for Illumina HiSeq paired end sequencing using Illumina’s TruSeq stranded RNA sample prep kit using polyA messenger RNA (mRNA) selection. mRNA was further processed directly including RNA fragmentation, first and second strand cDNA synthesis, adapter ligation and final library amplification following the manufacturer’s protocol. The final library was eluted in 30 µL elution buffer followed by quality assessment using a Bioanalyzer 2100 DNA1000 chip (Agilent Technologies) and quantified on a Qubit quantitation platform (Life Technologies).

Prepared libraries were pooled and diluted to 6 pM for TruSeq Paired End v4 DNA clustering on one single flow cell lane using a cBot device (Illumina). Final sequencing was done on an Illumina HiSeq 2500 platform using 126, 7, 126 flow cycles for sequencing paired end reads plus indexes reads. All steps for clustering and subsequent sequencing were carried out according to the manufacturer’s protocol. Reads were split per sample by using CASAVA 1.8 software (Illumina Inc, San Diego CA, USA). All sample preparations and sequencing were done by the Genomics lab of Wageningen University and Research, Business Unit Bioscience.

RNA poly-A enrichment library preparation and transcriptome sequencing (150 bp paired-end mode) on a NovaSeq 6000 platform of the second RNA sequencing experiment were conducted by Novogene UK Co. Ltd (Cambridge, UK).

First read quality was analyzed with FastQC (56) and MultiQC (57) packages in Python 2.7, followed by trimming of low quality reads with Trim Galore! (58). The reads were reexamined with FastQC and MultiQC after trimming and mapped to the *Arabidopsis* TAIR10 transcriptome (59) using Salmon (60). DESeq2 (61) package in R (62) was used for differential gene expression analysis and LFC shrinkage to correct for low transcript counts. Both timepoints were imported and analyzed separately in DESeq2. Heatmaps were created with the R package pheatmap (63), using Euclidean distance mapping and Ward.D clustering algorithms. GOseq (21) was used for GO enrichment analysis using GO_SLIM annotations from TAIR (version 2021-10-01). Enrichment was calculated using Fisher’s exact test with Benjamini–Hochberg (BH) correction for multiple testing. TF prediction was performed using the datafiles of PlantRegMap (17), a database containing both predicted regulation and experimental data. Enrichment was calculated using Fisher’s exact test with BH correction for multiple testing.

### Salt-Induced Tilting Assay (SITA).

*anac* mutants were screened for their sodium responses using SITA [as previously described (23)]. Growth conditions were as described above, but medium contained 0.5% sucrose. Seedlings were transferred to agar plates containing control or 100 mM NaCl and rotated 90° anticlockwise. Plates were imaged after 24 h with an Epson Perfection V850 Photo scanner. Root growth and angle were quantified after 24 h using Fiji with the SmartRoot (64) plugin.

### Halotropism.

Growth conditions as described above, but medium contained 0.5% sucrose. After five days, roots were aligned to be 5 mm from the cutting edge of the gradient, which was introduced by removing the lower part of the medium and replacing it with either ½ MS control or 200 mM NaCl medium. Plants were scanned at 24 and 48 h after the introduction of the gradient. ABA (5 mM stock in ethanol; Sigma) pretreated roots on a control (no NaCl) treatment had the tendency to grow away from the agar plate (n = 7, 15% at 24 h and n = 15, 33% at 48 h) and were excluded from quantification. This response was not observed for the ABA pretreated seedlings that received the NaCl treatment. After 48 h, plates were scanned with an Epson Perfection V800 at 400 dpi. Root growth and angle were quantified after 24 h using Fiji with the SmartRoot plugin (64). The timeseries was created by an automated infrared imaging setup, which has been described before (29, 65). Plants were grown in this growth chamber and were imaged after the introduction of the gradient. Roots were traced using an automated script (https://github.com/jasperlamers/timelapse-backtracing) as described before (29).

### Evans Blue Staining.

Whole seedlings were emerged in an Evans Blue solution (0.25% w/v, Sigma) for 15 min. Plants were destained for 15 min in sterile milliQ and the apical root was imaged with a DM2500 optical microscope (Leica) using a 10× objective. Images were stitched using pairwise stitching (66) in the Fiji software (67). The largest continuous stained area was segmented with an automated script in Python3.7 using OpenCV (v4.5.1.48; *SI Appendix*, Fig. S20).

### Root Diameter Analysis and RRTF1-mVenus Imaging.

Roots were treated for 1, 2 or 3 h (*pRRTF1::RRTF1-Venus*) or 24 h (root diameter) at 125 mM NaCl, followed by fixation using 4% paraformaldehyde and clearing using ClearSee (68). Roots were stained with CalcoFluorWhite and imaged on Stellaris 5 Confocal LSM (Leica) using a 10× dry lens. (excitation 405 nm, emission, 425 to 475 nm). Venus was excited at 515 m, and emission was detected between 525 to 550 nm.

### Hormone Measurements.

Hormone accumulation measurements were performed as described before, following the exact same procedure using root tissue rather than whole seedlings (69). In short, after 7 d, the plants were transferred to fresh agar plates supplemented with 125 mM NaCl (Duchefa), 125 mM KCl (Merck), 250 mM Sorbitol (Duchefa), or ½ MS control medium without supplements. After 1, 3, 6, and 12 h, roots were dissected with a sharp blade, weighed, flash frozen in liquid nitrogen and stored at −80 °C. Sixty roots (11.8 ± 3.0 mg material) were pooled in one biological replicate. Frozen material was ground to a fine powder using two stainless steel beads at 50 Hz for 1 min in a paint shaker (Fast & Fluid). Ground samples were extracted with 1 mL of 10% methanol containing 100 nM stable isotope-labeled internal standards for each investigated compound. Samples were extracted using an existing protocol with modifications (70). Namely, a StrataX 30 mg/3 mL SPE-column (Phenomenex) was used. Solvents were removed by speed vacuum system (ThermoSavant).

For detection and quantification by liquid chromatography-tandem mass spectroscopy, sample residues were dissolved in 100 µL acetonitrile /water (20:80 v/v) and filtered using a 0.2 µm nylon centrifuge spin filter (BGB Analytik). ABA was quantified by comparing retention times and mass transitions with ABA standards using a Waters XevoTQS mass spectrometer equipped with an electrospray ionization source coupled to an Acquity ultraperformance liquid chromatography (UPLC) system (Waters) as previously described (71, 72). Chromatographic separations were conducted using acetonitrile/water (+0.1% formic acid) on an Acquity UPLC BEH C18 column (2.1 mm × 100 mm, 1.7 µm, Waters) at 40 °C with a flowrate of 0.25 mL/min. First the column was equilibrated for 30 min using the solvent [acetonitrile/water (20:80 v/v) + 0.1% formic acid]. Samples were analyzed by injecting 5 µL, followed by the elution using program of 17 min in which the acetonitrile fraction linearly increased from 20% (v/v) to 70% (v/v). The column was washed after every sample by increasing the acetonitrile fraction to 100% in 1 min and maintaining this concentration for 1 min. The acetonitrile fraction was reduced to 20% in 1 min and maintained at this concentration for 1 min before injecting the next sample. The capillary voltage was set at 2.5 kV, the source temperature at 150 °C and the desolvation temperature at 500 °C. Multiple reaction monitoring was used for quantification (71). Parent–Daughter transitions and cone voltages were set using the IntelliStart MS Console. Peak quantification was processed with Targetlynx. Samples were normalized for the internal standard recovery and sample weight. All values were normalized to the 1-h control timepoint.

### Ion Measurements.

For the ion measurements of the ABA pretreatment, roots were harvested at 6 h after treatment and washed three times in milliQ water. The harvested material was dried for 24 h at 60°. The samples were measured using inductively coupled plasma mass spectrometer by the Nottingham Ionomics Facility (UK). All handling was done using plastic tweezers.

The ion measurements of the mutants (*aba3*, *snrk2.2/2.3,* and *rrtf1*) were done with X-ray fluorescence (XRF). Col-0, *snrk2.2/2.3, aba3, erf109-*1 seeds were surfaced-sterilized using 70% Ethanol for 1 min followed by 10 min of sterilization medium [30% of commercial bleach, 0.02% Triton X-100(Sigma)]. Then they were stratified in milliQ water in dark at 4 °C for 2 d. After stratification, they were sown on ½ MS medium including vitamins (Duchefa Biochemie), supplemented with 1.1 g/L MES (Duchefa Biochemie) and 1% Diashin Agar (Duchefa Biochemie), pH 5.8. and were then placed vertically at 90° in a growth chamber for 7 d in white light (100 µmol m^−2^ s^−1^ photosynthetic active radiation, 16 h photoperiod, 20 °C temperature). At day 7, seedlings were transferred to ½ MS with or without 125 mM NaCl for 6 h. Roots and Shoots samples were collected separately and rinsed with milliQ water. Samples were then dried at 60 °C for 1 wk. Dried tissues were then ground and measured using Zspec Elite (73).

### Statistical Analysis.

Single timepoint data were analyzed using Levene’s test to verify equal variances (*P* > 0.05) and Shapiro–Wilk to verify normal distribution (*P* > 0.05). Two-way ANOVA followed by a post hoc Tukey test when samples were normally distributed and had an equal variance. Alternatively, samples were analyzed using the nonparametric Dunn test preceded by a Kruskal–Wallis test (*P* < 0.05). BH was used to correct for multiple testing. Halotropism timeseries and root width were analyzed using nonpaired *t* tests per timepoint or length unit (NaCl vs. control per genotype), respectively, and corrected for multiple testing using the BH method. Other multi-timepoint data were analyzed per timepoint, using two-way ANOVA followed by the Tukey post hoc test [after Levene’s test to verify equal variances (*P* > 0.01) and Shapiro–Wilk to verify normal distribution (*P* > 0.05)]. All analyses were conducted in R and Microsoft Excel.

### Accession Numbers.

*RRTF1*, AT4G34410; *RAB18*, AT5G66400; *LTI78*/*RD29A*, AT5G52310; *SnRK2.2*, AT3G50500; *SnRK2.3*, AT5G66880; ABA2, AT1G52340; ABA3, AT1G16540; ABI1, AT4G26080; *PYR1*, AT4G17870; *PYL1*, AT5G46790; *PYL2*, AT2G26040; *PYL4*, AT2G38310, *ABI3*, AT3G24650; *ABI4*, AT2G40220; *ABI5*, AT2G36270; *AREB1*, AT1G45249; *AREB2*, AT3G19290; *AREB3*, AT3G56850; *ABF1*, AT1G49720; *RBOHC*, AT5G51060; *RBOHD*, AT5G47910; *RBOHF*, AT1G64060; *AOS*, AT5G42650; *JAR1*, AT2G46370; *MON1*, AT2G28390, *MOCA1,* AT5G18480.

## Supplementary Material

Appendix 01 (PDF)

Dataset S01 (XLSX)

Dataset S02 (XLSX)

Dataset S03 (XLSX)

Dataset S04 (XLSX)

Dataset S05 (XLSX)

Dataset S06 (XLSX)

Dataset S07 (XLSX)

## Data Availability

RNA seq Raw and processed RNA sequencing data files are publicly available via Array Express E-MTAB-13345 (74) for Fig.1 RNAseq, and E-MTAB-13709 (75) for Fig. 5 RNAseq. All other data are included in the article and/or supporting information.
