# Supplementary material for: Abscisic acid signaling gates salt-induced responses of plant roots
Source: Proc Natl Acad Sci U S A. 2025 Feb 5;122(6):e2406373122. doi: 10.1073/pnas.2406373122 (PMC11831169; doi:10.1073/pnas.2406373122)
Supplement: Supplementary file 1 — Appendix 01 (PDF) [file pnas.2406373122.sapp.pdf]

Supplemental Figures Lamers et al., 2025

Abscisic acid signaling gates salt-induced responses of plant roots

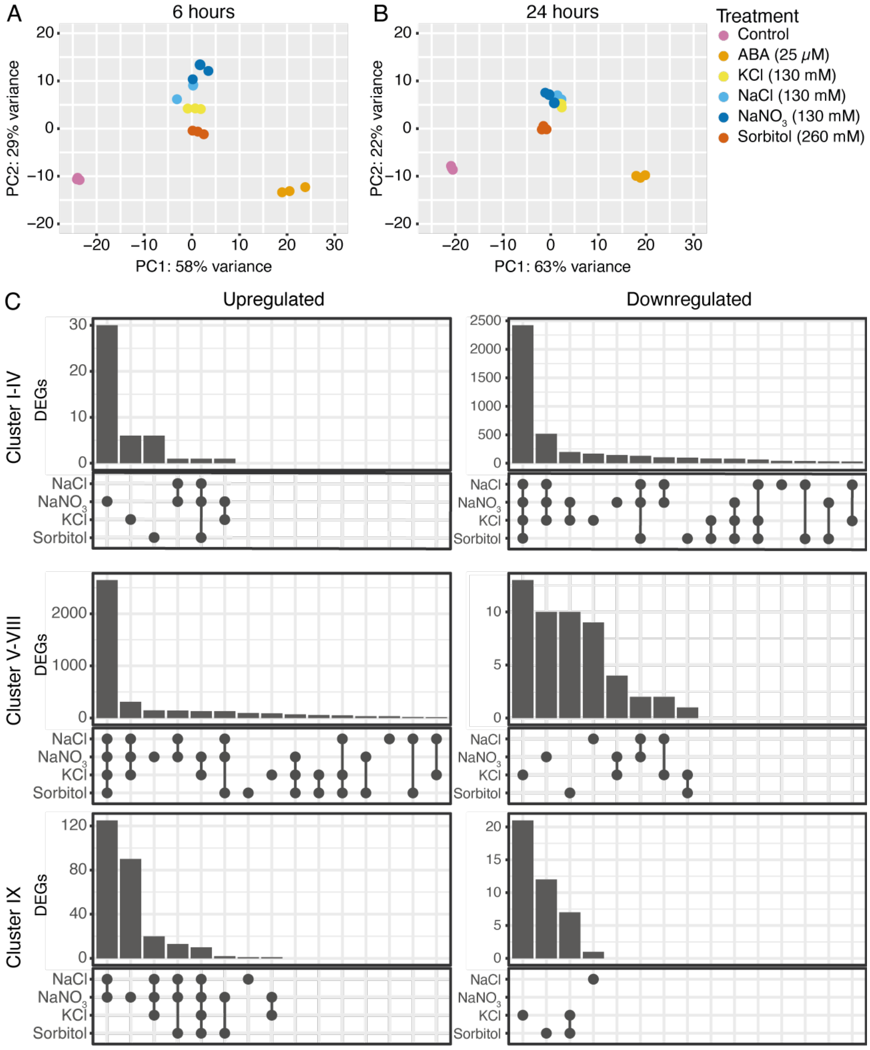

Figure S1. Stress-specific signaling in response to sodium ions is more apparent at 6 hours after treatment. (A-B) Principal component analysis (PCA) for normalized counts of all transcripts of root tissue of 8-day old seedlings treated for 6 or 24 hours. The included treatments were 130mM NaCl, 130mM $NaNO_3$, 130mM KCl, 260mM sorbitol and 25µM ABA on solid ½MS medium with 1g/L MES buffer. The first two components are presented. Colors indicate the different treatments. (A) 6 hours and (B) 24 hours after stress initiation. (C) The full upset plots of Fig. 1b. Here all intersecting DEGs (vs control) are shown per cluster pattern, up- (V-VIII) down- (I-IV) or sodium-induced (IX) regulation.

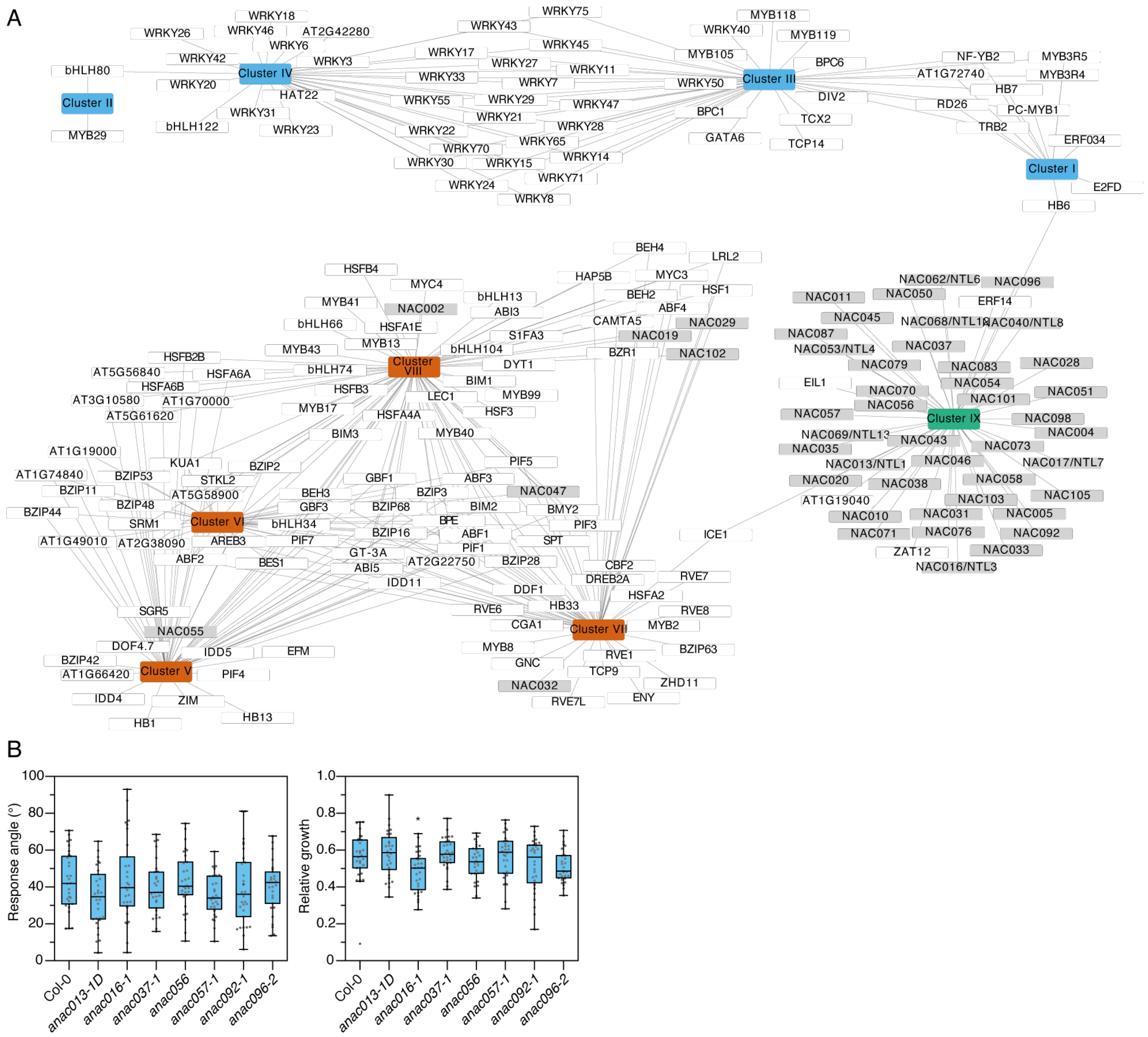

Figure S2. The sodium-specific gene expression cluster shows a distinct TF prediction pattern. (A) Enrichment of target genes per gene cluster (I-IX, see Figure 1A) for 766 TFs at 6 hours after stress induction using the datafiles of PlantRegMap[17]. The 216 significantly enriched TFs (FDR < 0.05) were mapped using Cytoscape 3.8.0[48]. The blue nodes are the downregulated clusters (I-IV), the red nodes the upregulated clusters (V-VIII), the green the sodium-specific cluster (IX) and the white nodes the TFs. NAC transcription factors are shaded. Connections indicate predicted TFs per cluster. 91 TFs were predicted for multiple clusters and thus have multiple connections. (Full data in Dataset S4). (B) Salt-induced-tilting assay (SITA) with mutants of NAC transcription factors. Plants were grown vertically for 4 days, followed by the transfer to 0mM control or 100mM NaCl agar plates. Plates were rotated 90 degrees. Both the root growth direction and growth were quantified after 24 hours. Response angle and relative growth between NaCl treatment and control treatment. Asterisks indicate significant differences from the wild-type (Dunn's test with Benjamini-Hochberg correction, $p < 0.05$, $n = 29\text{-}30$).

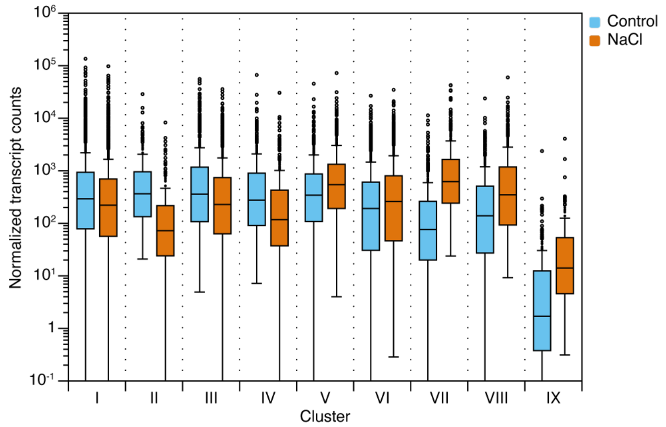

Figure S3. Genes in sodium-induced cluster IX are lowly expressed. Normalized transcript counts per cluster at 6 hours after stress induction in control and NaCl-treated samples.

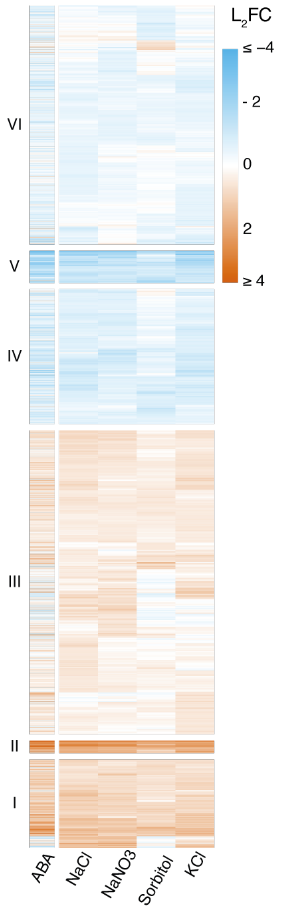

Figure S4. Osmotic and ionic stress and ABA-induced gene expression responses mostly overlap at 24 hours. Heatmap of all 5213 DEGs. Treatments (columns) and genes (rows) were clustered with Euclidean distance mapping and Ward clustering. ABA (25µM) was not used for clustering and was added afterwards as an annotation column. Colors indicate the $Log_2FoldChange$. The heatmap was divided into 6 clusters. The numbering is indicated left of the heatmap. (Full data in Dataset S5).

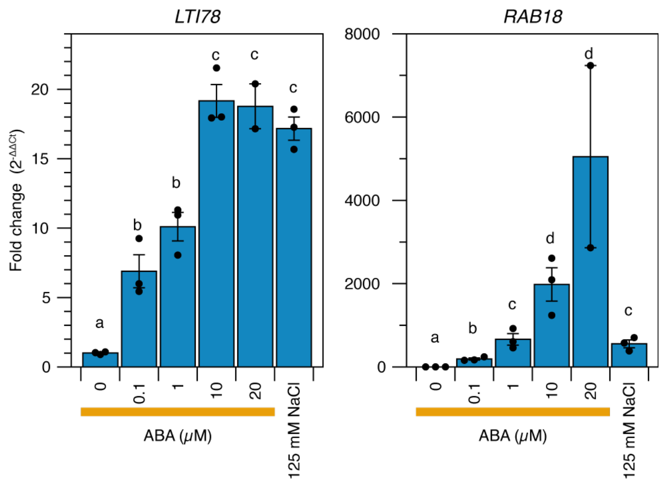

Figure S5. 125 mM NaCl-induced ABA marker gene expression corresponds to ABA-induced expression of a 1-10µM ABA treatment. Comparison of ABA-induced and NaCl-induced ABA marker gene expression (*LTI78* and *RAB18*) in 7-day-old Col-0 roots at 6 hours after the application of a range of ABA concentrations, or 125mM NaCl. Letters indicate significance (ANOVA + Tukey HSD, $p < 0.05$). Bars represent the mean values (n=2-4) +/- SE.

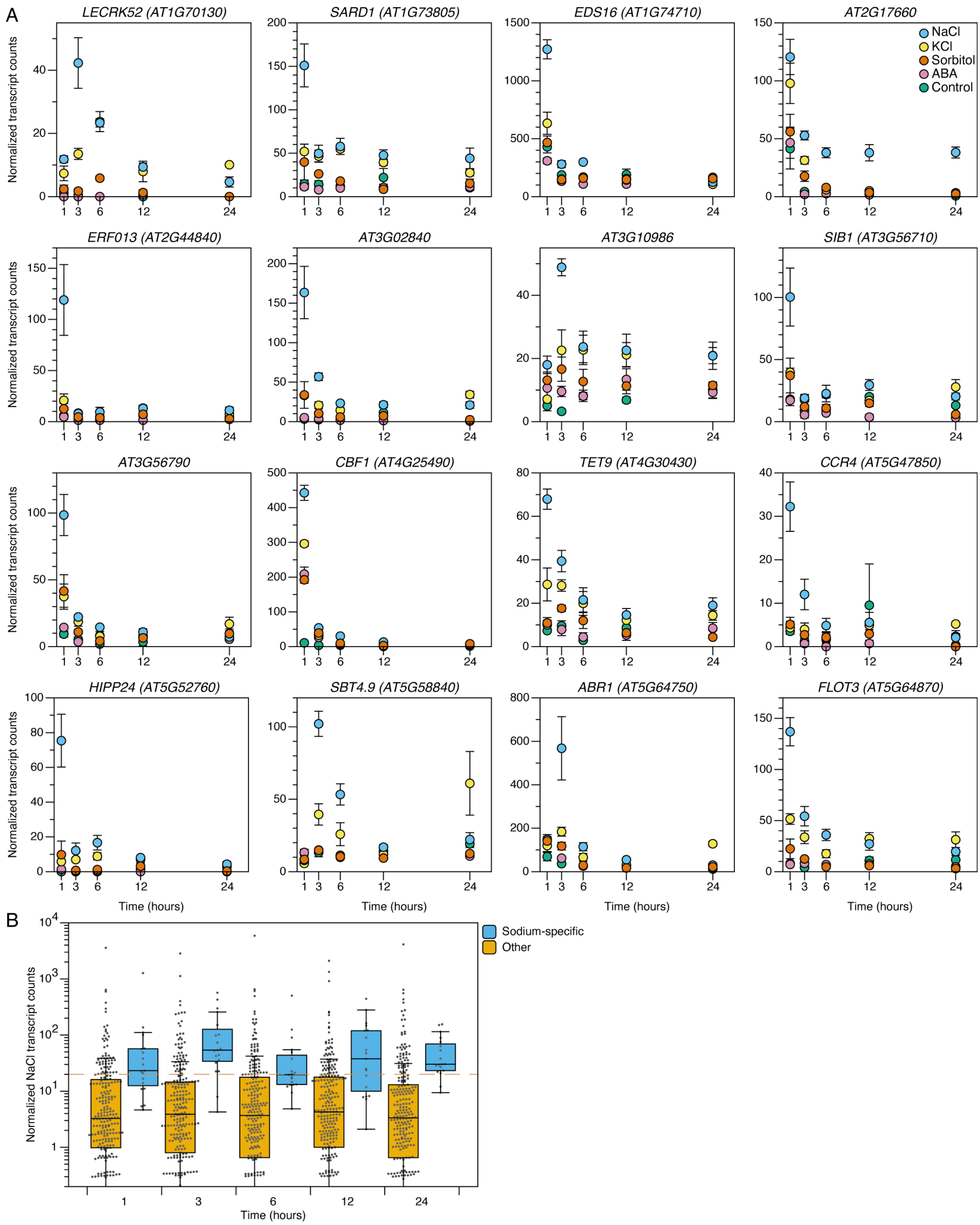

Figure S6. Sodium-induced gene expression is transient. (A) Normalized transcript counts over time for 16 sodium-induced genes in 7-day-old seedlings after transfer to control, 125 mM NaCl, 125 mM KCl or 250 mM sorbitol plates. Sodium-induced was defined as being significantly regulated at any timepoint by NaCl compared to all other treatments (control, KCl and sorbitol). In addition, genes were excluded if all NaCl samples had normalized transcript counts < 20. Dots represent the mean (n=3) +/- SE. Data was statistically analyzed using two-way ANOVA, followed by a Tukey post-hoc test. (B) Boxplots of normalized

transcript counts in the NaCl samples per timepoint of Cluster IX genes that were marked as sodium-induced in the second RNA sequencing experiment (n=20) or not (n=248).

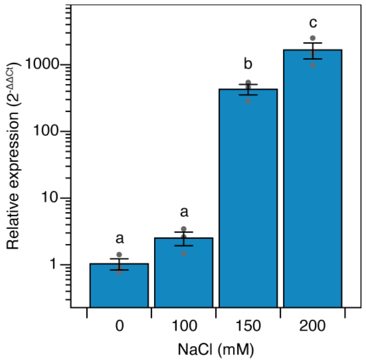

Figure S7. RRTF1 expression increases with NaCl concentration. *RRTF1* expression in 7-day-old Col-0 wildtype seedlings, transferred to 100, 150, 200 mM NaCl or control (1/2 MS) medium, and harvested after 1 hour. Data was statistically analyzed using one-way ANOVA, followed by a Tukey post-hoc test. Letters indicate significance ($p < 0.05$).

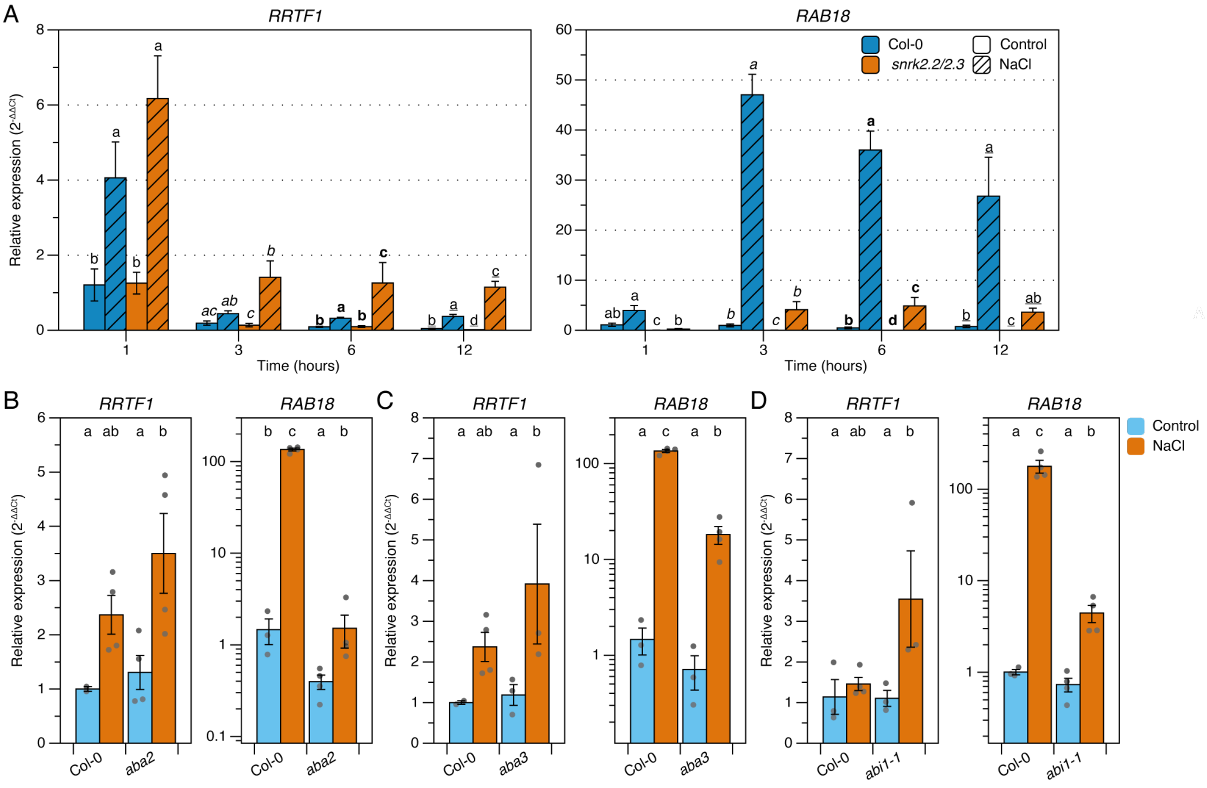

Figure S8. ABA biosynthesis and signaling mutants show enhanced *RRTF1* expression at 3 hours after salt treatment onwards. (A) *RRTF1* and *RAB18* expression in 7-day-old *snrk2.2/2.3* and wild type seedlings, transferred to 125 mM NaCl or control (1/2 MS) medium, and harvested after multiple timepoints. Levels are normalized for control conditions at 1 hour. Data was statistically analyzed using two-way ANOVA per timepoint, followed by a Tukey post-hoc test. Letters of the same font indicate significance ($p < 0.05$). (B-D) *RRTF1* and *RAB18* expression in 7-day-old ABA biosynthesis mutants *aba2* (B) and *aba3* (C) and ABA signaling mutant *abi1-1* (D) at 1 hour after stress induction. Data was statistically analyzed using two-way ANOVA per timepoint, followed by a Tukey post-hoc test. Letters indicate significance ($p < 0.05$).

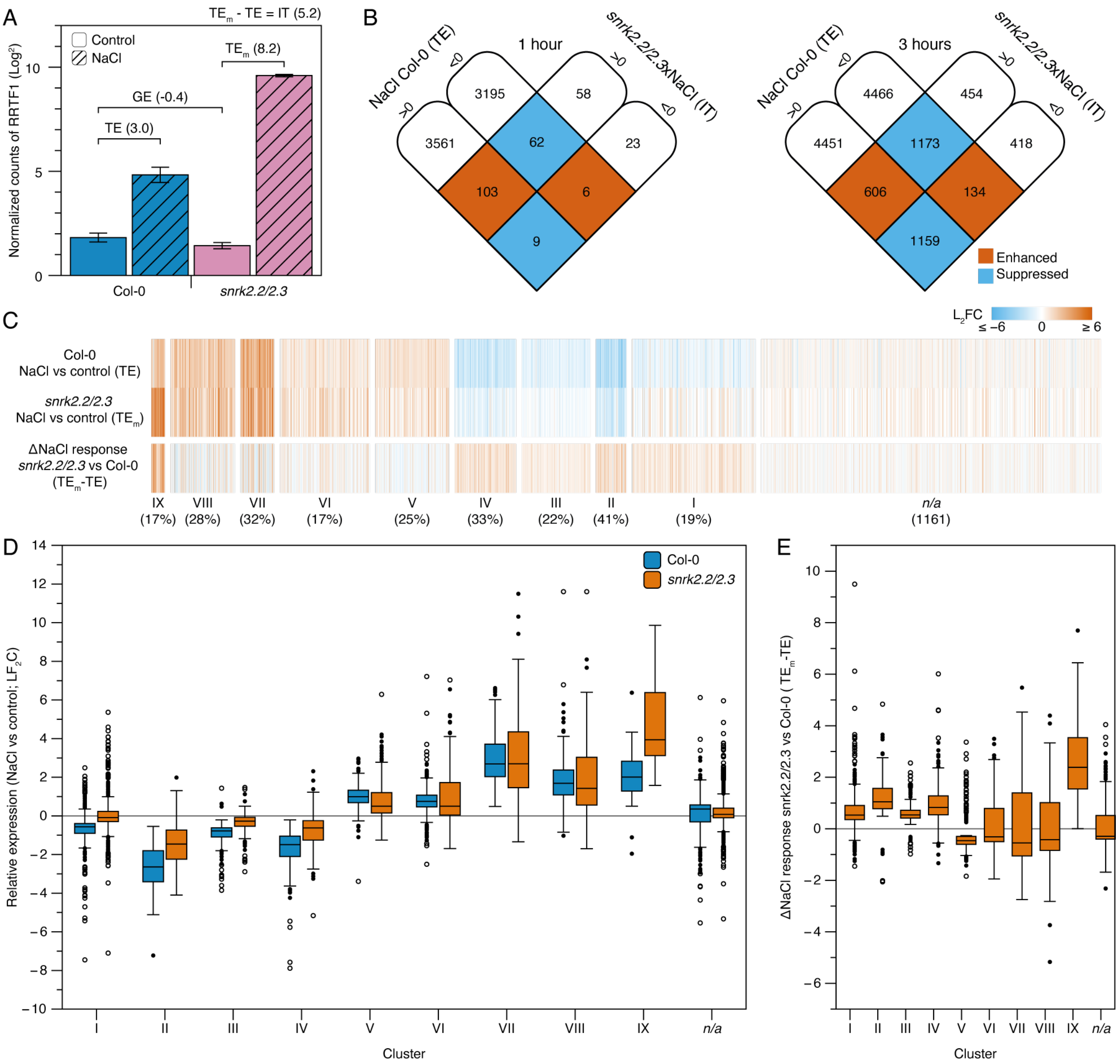

Figure S9. The ABA-insensitive *snrk2.2/2.3* mutant shows enhanced sodium-induced transcriptional responses. (A) Example of normalized counts of *RRTF1* in *snrk2.2/2.3* and wild type Col-0 at 3 hours. Different outputs of the DESeq2 software are indicated. Treatment effect (TE; response to a treatment in wildtype), Genotype effect (GE; difference between mutants and wildtype in control conditions) or Interaction term (IT; differences in response to a treatment between a mutant and wildtype). The IT is the difference between the treatment effect of the mutant (TE_m) and the TE. Bars represent mean values +/- SE (n=3). (B) The overlap between the *snrk2.2/2.3*xNaCl IT with NaCl and the NaCl TE (Col-0) for up- and downregulated DEGs (FDR < 0.05) at 1 and 3 hours after stress induction. Colors indicate enhancement or suppression. (Full data in Dataset S6). (C) The full heatmap of Fig. 3B including the genes that were identified in the RNA sequencing experiment of Fig. 3, but not in the experiment of Fig. 1. (D-E) Boxplot of the expression data in the heatmap of panel C. (D) Relative expression (NaCl vs control) of *snrk2.2/2.3* and Col-0 per cluster. (E) The interaction factor of *snrk2.2/2.3* per cluster (*i.e.*the difference in the treatment effect (TE_m-TE) for *snrk2.2/2.3* vs Col-0).

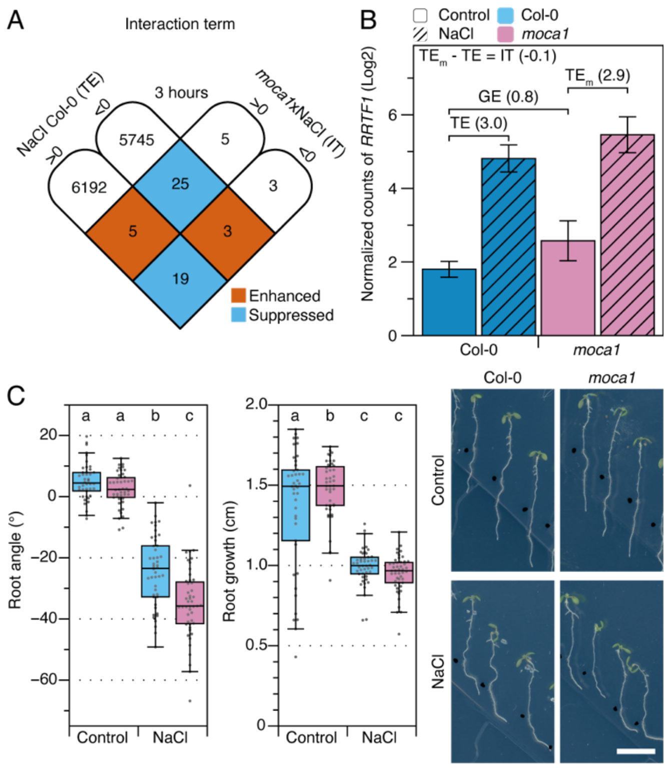

Figure S10. The *moca1* mutant is not impaired in halotropism, and shows very few differential transcriptional responses during NaCl stress. (A) The overlap between the *moca1*xNaCl IT with NaCl and the NaCl TE (Col-0) for up- and downregulated DEGs (FDR < 0.05) at 3 hours after stress induction. Colors indicate enhancement or suppression. (B) Normalized counts of *RRTF1* in Col-0 and *moca1*. Different outputs of the DESeq2 software are indicated. Treatment effect (TE; response to a treatment in wildtype), Genotype effect (GE; difference between mutants and wildtype in control conditions) or Interaction term (IT; differences in response to a treatment between a mutant and wildtype). The IT is the difference between the treatment effect of the mutant (TE_m) and the TE. Bars represent mean values +/- SE (n=3). (C) Quantification and representative images of the root growth angle and root growth (NaCl/control) of 7-day old Col-0 and *moca1* seedlings at 48 hours after the introduction of the NaCl gradient (n=40-48). Letters indicate significant differences after Dunn test ($p < 0.05$) and corrected for multiple testing using Benjamin-Hochberg (BH) procedures.

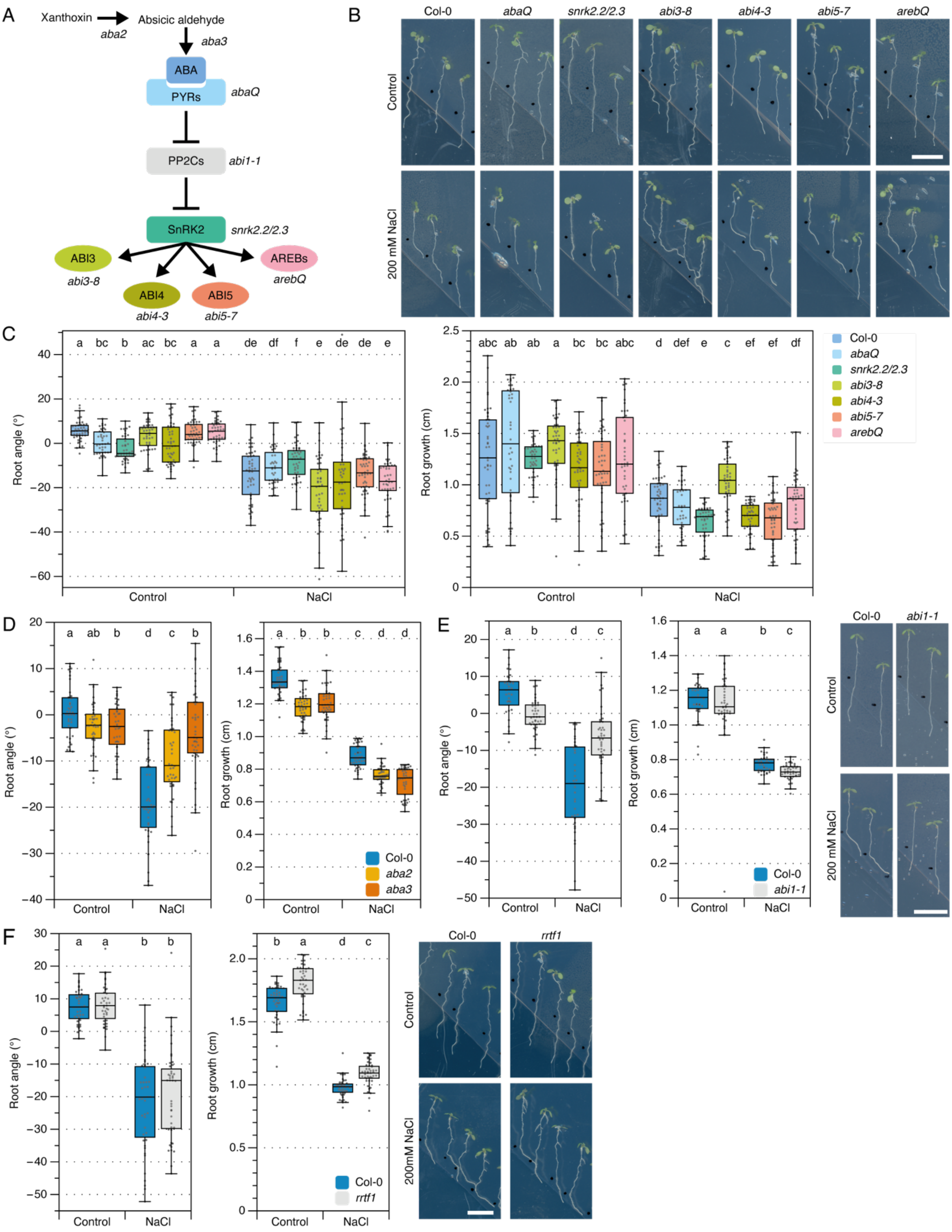

Figure S11. ABA biosynthesis and signaling mutants show a reduced halotropic response. (A) Simplified representation of the ABA biosynthesis and signaling pathways. ABA is metabolized from xanthoxin to abscisic aldehyde by ABA2 and from abscisic aldehyde to ABA by ABA3. ABA is sensed by *PYRABACTIN RESISTANCE1 (PYR)/PYR1-LIKE* family of receptors, which releases the inhibition by PROTEIN PHOSPHATASES TYPE 2C (PP2C) proteins of the SUCROSE NON-FERMENTING 1-RELATED PROTEIN KINASE2 family (SnRK2.2). SnRK2s activate downstream transcription factors (TFs) such as ABA INSENSITIVE 3 (ABI3),

ABI4, ABI5 and ABA-RESPONSIVE ELEMENT-BINDING FACTOR/ABSCISIC ACID RESPONSIVE ELEMENT-BINDING FACTOR 1 TFs (AREB/ABF). (B-C) The complete dataset as shown in Fig. 4B. (B) Representative images of the halotropism assay. (C) Absolute data of the halotropism assay of 7 day-old seedlings at 48 hours after the introduction of the gradient. Statistical analysis was performed using non-parametrical Dunn's test and corrected for multiple testing using Benjamin-Hochberg (BH) procedures (n = 35 – 48). Letters indicate statistical groups. (D-F) Quantification and representative images of the root growth angle and root growth of 7-day old seedlings at 48 hours after the introduction of the NaCl gradient (n=40-48). Letters indicate significant differences after Dunn test ($p < 0.05$) and corrected for multiple testing using Benjamin-Hochberg (BH) procedures. (D) The absolute data as shown in Fig. 4A. (E) The ABA signaling mutant *abi1-1*. (F) The mutant of the robustly sodium induced expression marker RRTF1.

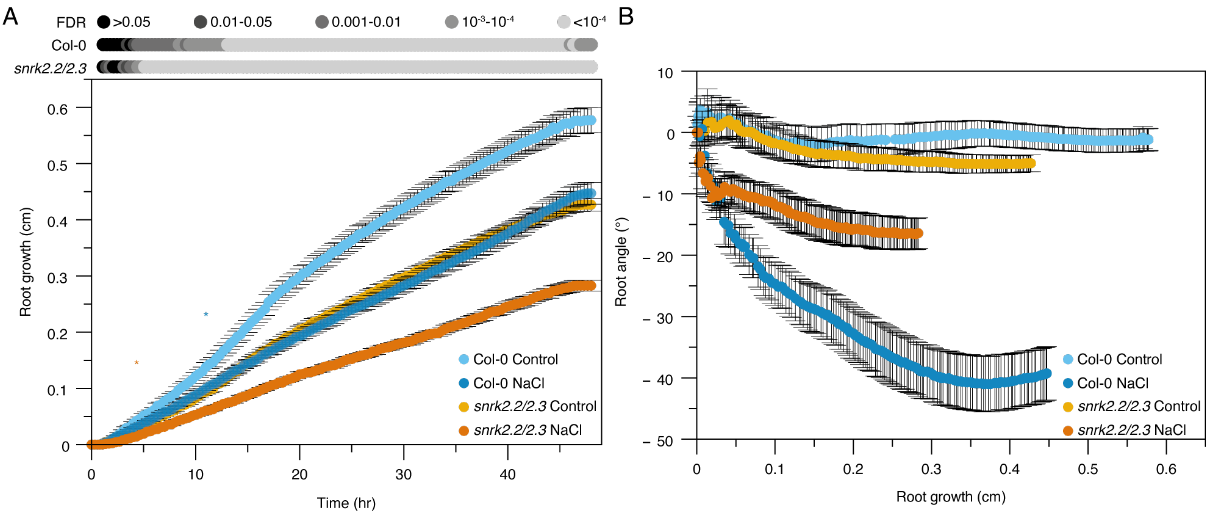

Figure S12. The halotropism phenotype of *snrk2.2/2.3* is not caused by the reduced root growth. (A) Root growth of the halotropism timelapse data shown in Fig. 4C. Seedlings were imaged every 20 minutes for 48 hours. Dots represent mean values (n=20) +/- SE. Pairwise Welch tests were performed for NaCl treated samples and the control condition of the same genotype and corrected for multiple testing with BH. Corrected p-values (NaCl vs control) are shown in grey-values above the graph. (B) Timelapse data as shown in Fig. 4C, plotted with the angle against the length.

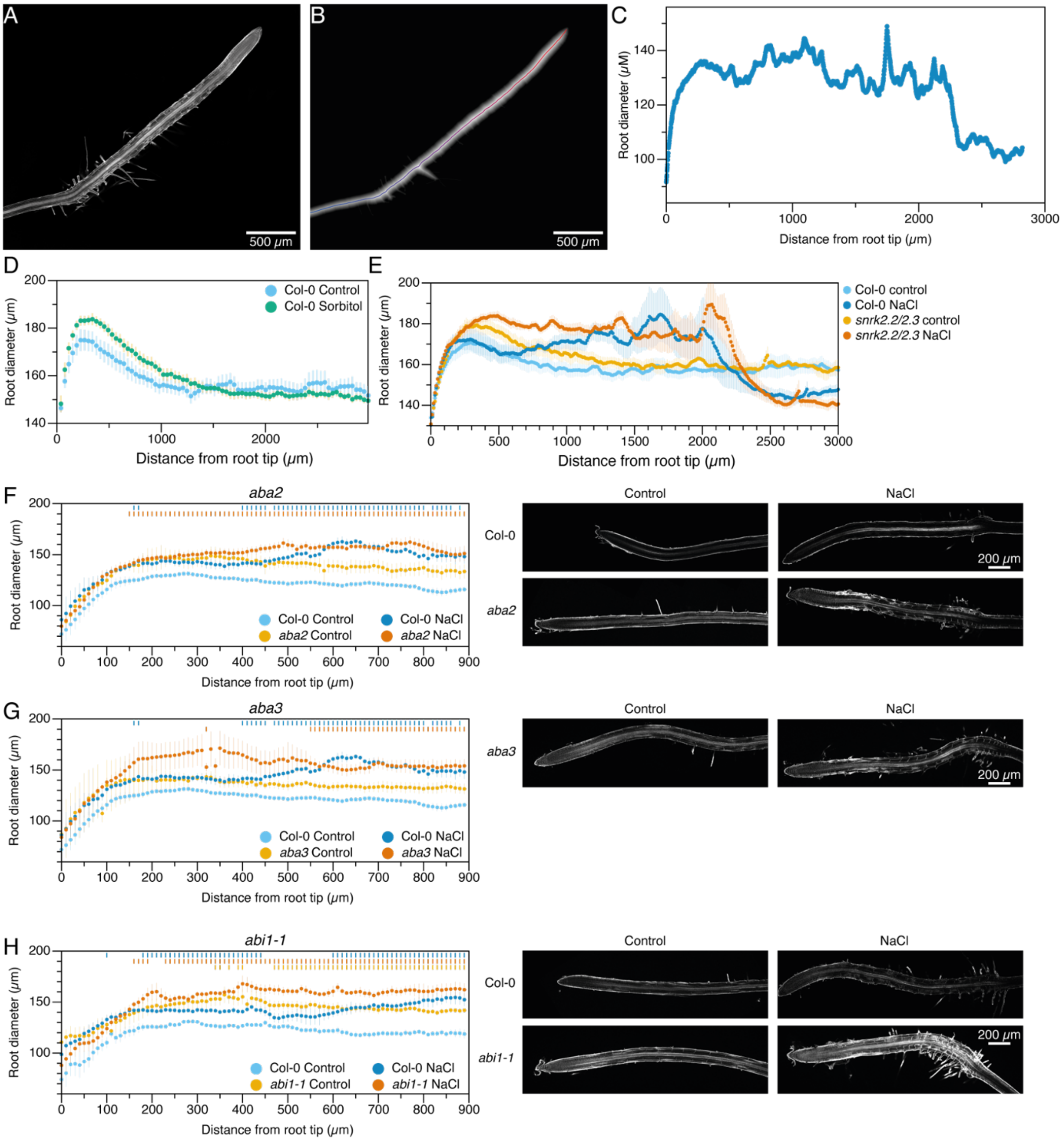

Figure S13. Root diameter is increased by NaCl but not by osmotic stress. (A) Confocal image of a NaCl-treated root fixed at 24 hours after stress induction and counterstained with Calcofluor white. (B) Euclidean distance map of a segmented image. Roots were segmented based on pixel values, followed by calculating the distance of each root-pixel to the closest non-root pixel (indicated by gray intensity). The midline was calculated using this distance map, filtering for the highest distance-values in an area of 5x5 and connecting these filtered pixels together. The midline is indicated from red to blue (red = root tip). Next, the Euclidean distance values of every point on the midline were used as root diameter. (C) Plot of the root diameter along the midline. (D) Root diameter along the root axis (length 0 = root tip) for Col-0 with control or 250mM Sorbitol treatment. No significant differences were observed using multiple Welch tests followed by BH correction for multiple testing (Sorbitol vs control). (n = 10). (E) Quantification of root diameter ratio along the root axis (length 0 = root tip) for Col-0 and *snrk2.2/2.3*. The region of 0-1400 µm is shown in Fig. 4E. NaCl induced cell swelling from the root tip till 2200 µm from the root tip. Older cells of the root were shrunken by the salt

treatment (2200-3000 µm). (F-H) Quantification of the root diameter along the root axis of *aba2*, *aba3* and *abi1-1* mutants. The blue bars above the graph indicate significant differences (FDR < 0.05, n=3-5) between NaCl vs control treated Col-0 roots as analyzed using multiple Welch tests followed by BH correction for multiple testing. The red bars indicate significant differences between NaCl treated mutant roots and Col-0 roots. The orange bars the differences between wildtype and mutant under conditions. Confocal images show calcofluor-white stained roots at 24 hours after NaCl treatment.

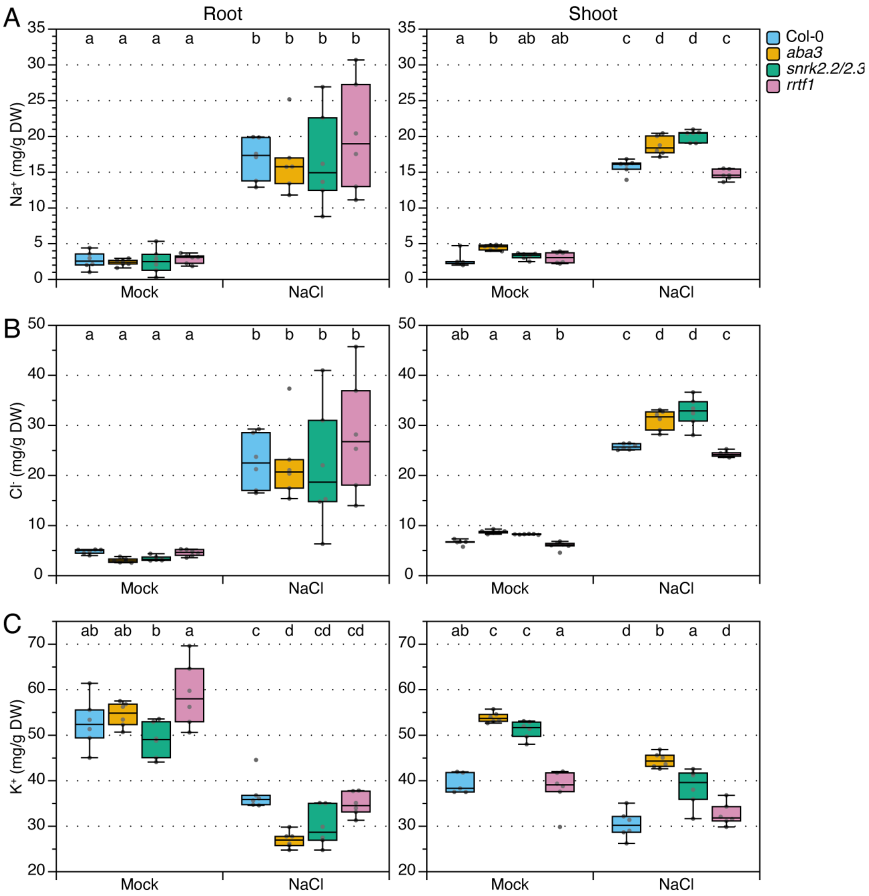

Figure S14. Shoots of ABA biosynthesis and signaling mutants accumulate more Na^+ and Cl^- during NaCl stress. Sodium, chloride and potassium contents in roots and shoots of 7-day-old Col-0 wildtype, *aba3*, *snrk2.2/2.3* or *rrtf1* seedlings after transfer to control or 125 mM NaCl medium for 6 hours. Letters indicate significant differences by two-way ANOVA followed by a Tukey post hoc ($p < 0.05$, n = 5-6).

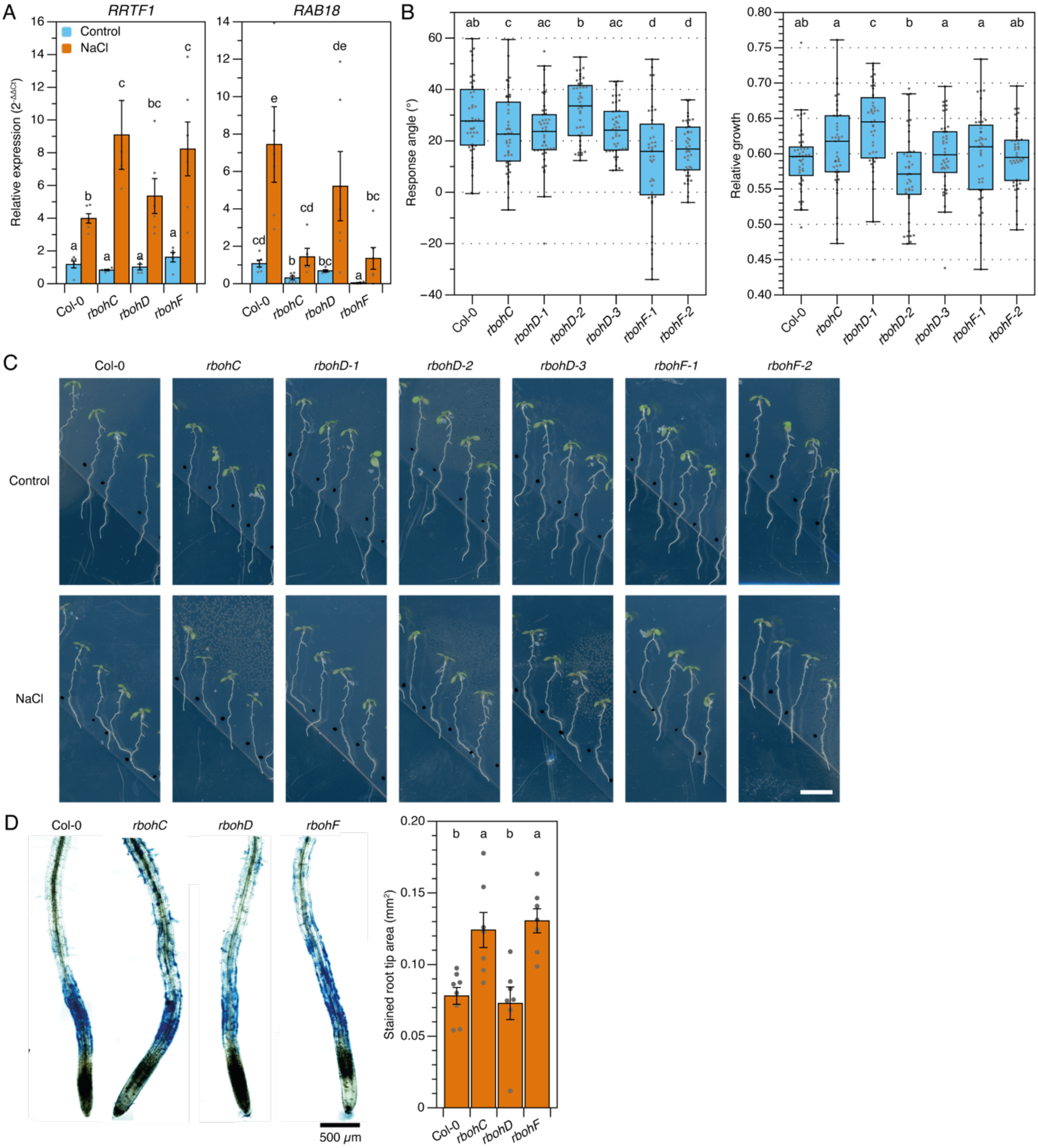

Figure S15. Extracellular ROS production is important for halotropism. (A) qPCR analysis of sodium-induced *RRTF1* transcripts in roots of 7-day old JA biosynthesis mutants and Col-0 control. Seedlings were treated with control or 125 mM NaCl for one hour. Bars represent mean values +/- SE, n = 5 and individual datapoints are shown as dots. Letters indicate significant differences by two-way ANOVA followed by a Tukey post hoc ($p < 0.05$). (B-C) Quantification and representative images of the halotropism response angle and relative growth of *rbobc* (1 allele)*, rbobd* (3 alleles) and *rbobf* (2 alleles) mutants and Col-0 control at 48 hours after the introduction of the NaCl gradient (introduced when seedlings were 5-day-old. Letters indicate significant differences after Dunn's test ($p < 0.05$, n=42-45). (D) Representative images of the cell damage assay by Evans Blue staining *rboh* mutants and Col-0 after NaCl-stress for 48 hours. (E) The affected area of the assay (D) was quantified using a script (Fig. S15). Bars represent mean values +/- SE, n=7 - 8 and individual datapoints are

shown as dots. Letters indicate significant differences by two-way ANOVA followed by a Tukey post hoc ($p < 0.05$).

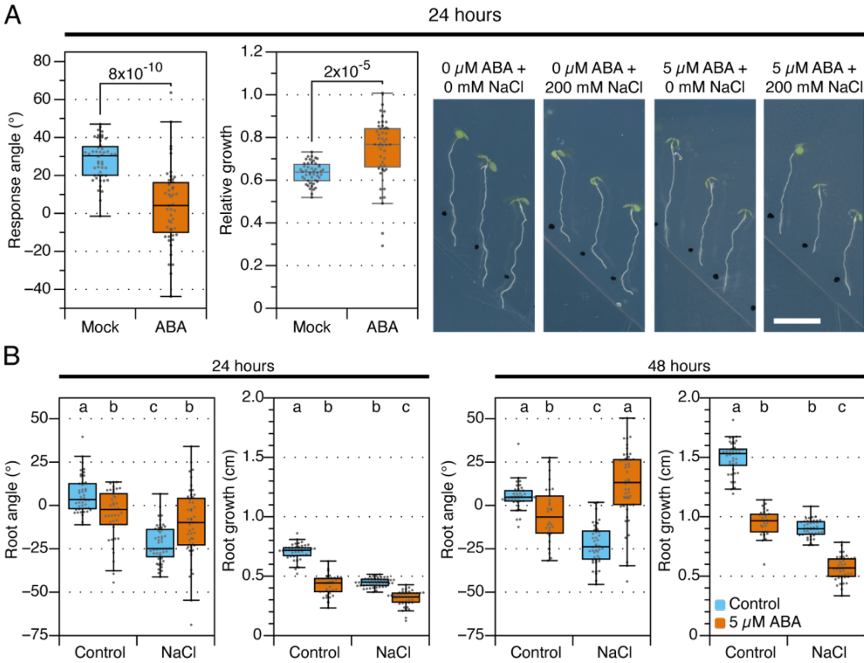

Figure S16. ABA-pretreatment represses halotropism. (A) Representative images and quantification of the halotropism response with or without a 12-hour ABA pretreatment in 5-day-old seedlings at 24 hours after the introduction of the gradient. The relative growth shows the ratio between NaCl and control conditions for the respective ABA pretreatment. Statistics were performed using pairwise t-tests. (B) Absolute data of the halotropism with ABA-pretreatment at 24 and 48 hours after introduction of the gradient (As shown in Fig. 5B). Different letters indicate significant differences after Dunns test with BH correction for multiple testing ($p < 0.05$). At 24 hours, (n=40-48) and at 48 hours (n=30-48).

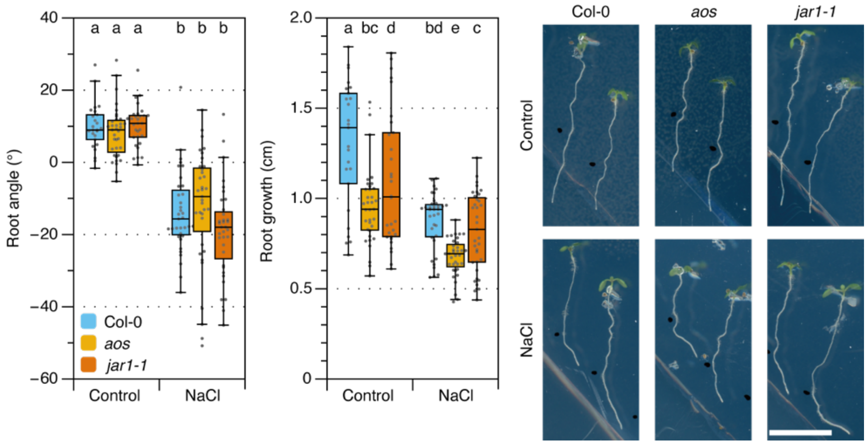

Figure S17. RRTF1-Venus is present at 2 and 3 hours after stress induction. Maximum projection confocal images the same root expressing *pRRTF1::RRTF1-Venus* at 2 and 3 hours after 125 mM NaCl treatment. RRTF1-Venus was not visible at 1 hour after stress treatment.

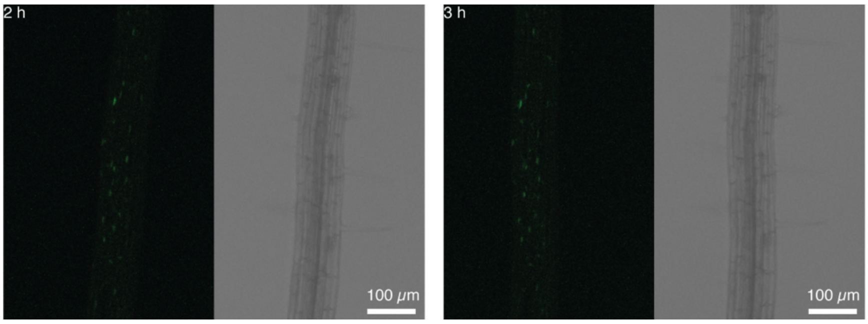

Figure S18. Sodium-specific responses are not JA-dependent. Absolute data of the halotropism assay shown in Fig. 6C. Quantification of the halotropism response angle and relative growth of the main root at 48 hours after the introduction of the NaCl gradient (introduced when seedlings were 5-days-old). JA signaling mutants (*aos* and *jar1-1*) and Col-0 control (n=35). Letters indicate significant differences after Dunns test with BH correction for multiple testing ($p < 0.05$).

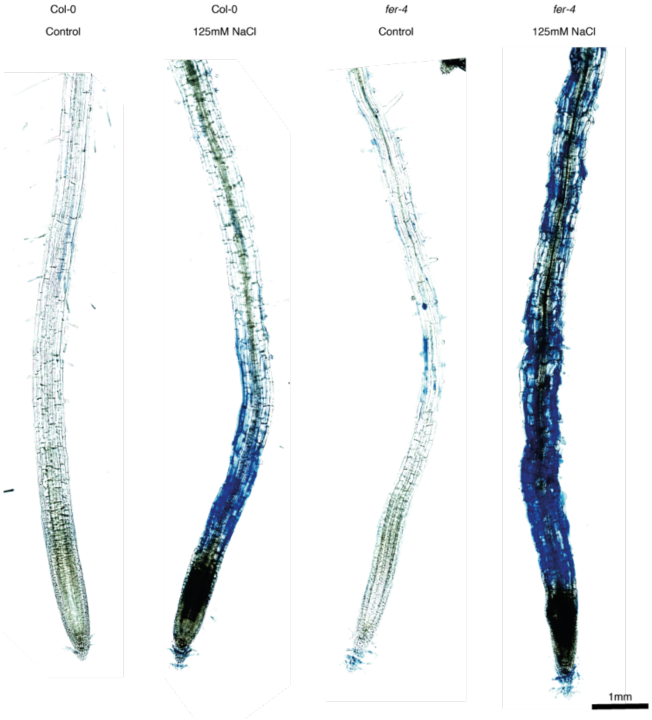

Figure S19. The *feronia-4* (*fer-4*) mutant has increased plasma permeability. Representative images of the Evans Blue cell damage assay of the *fer-4* mutant and Col-0 after NaCl-stress for 48 hours.

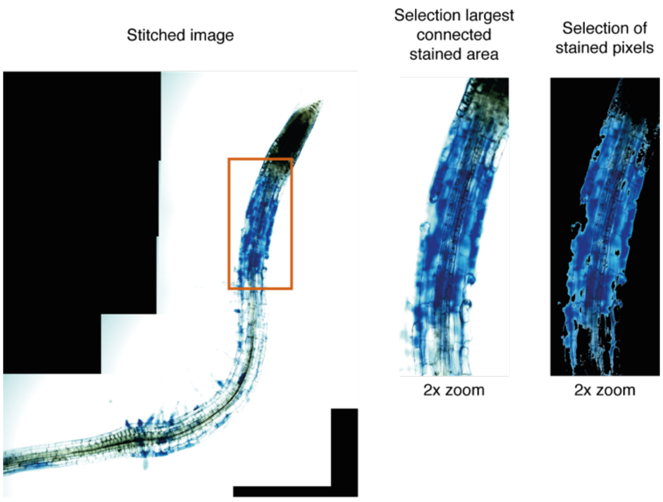

Figure S20. Evans Blue staining was analyzed with a script. Automized script to analyze the Evans Blue staining. First pixels are filtered to be dominantly blue (in an RGB channel), followed by the selection of the largest continuous blue object and all object in close vicinity (displayed in the 2nd panel). Finally, all blue pixels in this selected area are counted (displayed in the 3rd panel).
